# The lung microbiome, peripheral gene expression, and recurrence-free survival after resection of stage II non-small cell lung cancer

**DOI:** 10.1186/s13073-022-01126-7

**Published:** 2022-10-27

**Authors:** Brandilyn A. Peters, Harvey I. Pass, Robert D. Burk, Xiaonan Xue, Chandra Goparaju, Christopher C. Sollecito, Evan Grassi, Leopoldo N. Segal, Jun-Chieh J. Tsay, Richard B. Hayes, Jiyoung Ahn

**Affiliations:** 1 Department of Epidemiology and Population Health, Albert Einstein College of Medicine, 1300 Morris Park Avenue, #1315AB, The Bronx, New York, NY 10461 USA; 2 Department of Cardiothoracic Surgery, NYU Langone Health, New York, NY USA; 3 NYU Perlmutter Cancer Center, New York, NY USA; 4 Department of Pediatrics, Albert Einstein College of Medicine, The Bronx, New York, NY USA; 5 Department of Microbiology & Immunology, and Obstetrics & Gynecology & Women’s Health, Albert Einstein College of Medicine, The Bronx, New York, NY USA; 6 Department of Medicine, NYU Langone Health, New York, NY USA; 7 Department of Population Health, NYU Langone Health, New York, NY USA

**Keywords:** Lung microbiome, Lung cancer, Peripheral blood, Gene expression, Recurrence, Survival

## Abstract

**Background:**

Cancer recurrence after tumor resection in early-stage non-small cell lung cancer (NSCLC) is common, yet difficult to predict. The lung microbiota and systemic immunity may be important modulators of risk for lung cancer recurrence, yet biomarkers from the lung microbiome and peripheral immune environment are understudied. Such markers may hold promise for prediction as well as improved etiologic understanding of lung cancer recurrence.

**Methods:**

In tumor and distant normal lung samples from 46 stage II NSCLC patients with curative resection (39 tumor samples, 41 normal lung samples), we conducted 16S rRNA gene sequencing. We also measured peripheral blood immune gene expression with nanoString®. We examined associations of lung microbiota and peripheral gene expression with recurrence-free survival (RFS) and disease-free survival (DFS) using 500 × 10-fold cross-validated elastic-net penalized Cox regression, and examined predictive accuracy using time-dependent receiver operating characteristic (ROC) curves.

**Results:**

Over a median of 4.8 years of follow-up (range 0.2–12.2 years), 43% of patients experienced a recurrence, and 50% died. In normal lung tissue, a higher abundance of classes Bacteroidia and Clostridia, and orders Bacteroidales and Clostridiales, were associated with worse RFS, while a higher abundance of classes Alphaproteobacteria and Betaproteobacteria, and orders Burkholderiales and Neisseriales, were associated with better RFS. In tumor tissue, a higher abundance of orders Actinomycetales and Pseudomonadales were associated with worse DFS. Among these taxa, normal lung Clostridiales and Bacteroidales were also related to worse survival in a previous small pilot study and an additional independent validation cohort. In peripheral blood, higher expression of genes TAP1, TAPBP, CSF2RB, and IFITM2 were associated with better DFS. Analysis of ROC curves revealed that lung microbiome and peripheral gene expression biomarkers provided significant additional recurrence risk discrimination over standard demographic and clinical covariates, with microbiome biomarkers contributing more to short-term (1-year) prediction and gene biomarkers contributing to longer-term (2–5-year) prediction.

**Conclusions:**

We identified compelling biomarkers in under-explored data types, the lung microbiome, and peripheral blood gene expression, which may improve risk prediction of recurrence in early-stage NSCLC patients. These findings will require validation in a larger cohort.

**Supplementary Information:**

The online version contains supplementary material available at 10.1186/s13073-022-01126-7.

## Background

Lung cancer remains the leading cause of cancer death worldwide [1] and in the USA [2], with non-small cell lung cancer (NSCLC) and its two primary sub-types, lung adenocarcinoma and squamous cell carcinoma, accounting for the majority of lung cancers [3]. In patients with early-stage (I to II) NSCLC, surgical resection with or without cytotoxic chemotherapy is the primary mode of curative treatment [2], but cancer recurrence, a strong predictor for lung cancer mortality, is common: 5-year survival rates range from 83% for stage IA to 53% for stage IIB [4]. Advancements in molecularly targeted therapies and immunotherapies, usually applied to later-stage NSCLC, have led to improvements in survival over the past two decades; there is a great need for developing biomarkers predicting for recurrence in early-stage NSCLC [3]. The field has predominantly focused on biomarkers from the tumor environment, such as mutational burden [5], immune cell infiltration [6], and local gene expression [7–11], yet novel biomarkers from the human microbiota and peripheral immune environment are understudied and may hold promise for prediction as well as improved etiologic understanding of lung cancer recurrence.

Recently, the lung microbiome has emerged as a potential modulator of lung cancer development and recurrence [12]. While the high biomass gut microbiome has received much attention for its role in anti-cancer immunosurveillance and immunotherapy response [13], the low biomass community of microorganisms resident in the human lung may serve a locally important homeostatic role, providing immune surveillance and protection from pathogens [14]. A dysbiotic lung microbiome related to lung disease may promote inflammation and exacerbate lung disorders, including lung cancer [15]. In a mouse model of lung adenocarcinoma, lung microbiota drove activation of lung γδ T-cells, which in turn promoted neutrophil infiltration and tumor cell proliferation, while germ-free and antibiotic-treated mice were protected from lung cancer [16]. In human studies, composition of the lower airway microbiome has been associated with NSCLC prognosis [17, 18], as well as pulmonary inflammation and carcinogenic transcriptomic pathways [19, 20]. We have previously reported an association of normal lung tissue microbiome composition with NSCLC recurrence in a small (*n* = 17) pilot study [21]. As research on the lung microbiome and recurrence is in its infancy, the availability of only a few studies with heterogeneous methods and populations has precluded yet identifying common bacterial signatures associated with prognosis.

The peripheral immune system is crucial for building antitumor immune responses, including those induced by immunotherapy [22]. Many cancers, including NSCLC, drive perturbations in systemic immune organization [22] and peripheral blood immune cell (PBMC) phenotypes have been associated with responses to chemotherapy and immunotherapy in later-stage NSCLC [23, 24]. In principle, peripheral immunity also plays a role in the recurrence of early-stage NSCLC, but few studies have explored this hypothesis. Higher preoperative lymphocyte/monocyte ratio [25] and reduced neutrophil/lymphocyte ratio [26] have been associated with improved survival in early-stage NSCLC. Additionally, a PBMC gene expression signature was previously shown to predict survival in early-stage NSCLC [27, 28].

Here, we examine tumor and normal lung tissue microbiota and peripheral blood gene expression as predictors of recurrence in stage II NSCLC. We aimed to identify novel biomarkers that may provide mechanistic insights into recurrence for early-stage NSCLC, and/or be useful in recurrence risk prediction.

## Methods

### Patients and sample collection

Samples were selected from the NYU Thoracic Surgery Archives (NTSA). Established in 2006, the NTSA has prospectively collected serum, plasma, buffy coat, and peripheral blood mononuclear cells, along with lung cancer and matching normal lung specimens under the IRB-approved 8896 protocol. Patients identified on preoperative workup as having a pulmonary nodule suspicious for lung cancer were consented for the collection of blood and snap frozen tissues (tumor and remote lung from the same lobe/segment) in the operating room at the time of their resection. Lung and matching tumor are sterilely cut at the operating room table, transferred to pre-labeled Nunc^TM^ vials and immediately snap frozen in liquid nitrogen within 10 min of resection. Samples are de-identified for storage at −80°C until use.

Demographic, clinical, and pathological data are recorded in an encrypted Research Electronic Data Capture (REDCap) database. Patients are seen at 3-month intervals for 2 years, then at 6-month intervals for 1 year, and then annually, with CT scans performed for surveillance in order to document any systemic and loco-regional recurrences, or the development of a second primary tumor. For this study, 48 patients with stage II NSCLC and >3 tumor and normal lung sample aliquots available in the NTSA were selected for microbiome measurement. We excluded patients with <1-month survival after resection, resulting in inclusion of 46 patients (Supplementary Figure 1). For microbiome analysis, we excluded samples with low microbial sequence reads (explained in further detail below), resulting in 39 tumor samples and 41 normal lung samples (34 patients with both tumor and normal lung samples) (Supplementary Figure 1). For peripheral gene expression analysis, we excluded 3 patients missing a buffy coat sample and 1 additional patient with low gene expression counts, leaving 42 patient samples (Supplementary Figure 1).

### Outcome definitions

Endpoints were defined according to the consensus agreement in Punt et al. [29]. Disease-free survival (DFS) includes recurrences (loco-regional and systemic), new primaries (same or other cancer), and death from any cause as events. Recurrence-free survival (RFS) includes recurrences (loco-regional and systemic) and death from any cause as events, ignoring new primaries as events. Overall survival (OS) includes death from any cause as events. For all endpoints, person-time is defined as time from surgery to event or loss to follow-up (censored). For two patients missing the date of death, date of recurrence was assigned as the date of death; sensitivity analyses excluding these patients did not substantially change our findings.

### Covariates

Data available for these patients were age (years), sex (male, female), race (white, non-white), smoking status (never, former, current), histology (adenocarcinoma, squamous cell carcinoma), stage (IIA, IIB), lymphovascular invasion (yes, no), pleural invasion (yes, no), tumor size (cm), positive nodes (%), and chemotherapy (yes, no). Variables associated with RFS, DFS, or OS in unadjusted or multivariable Cox proportional hazards models (*p*<0.10) were included in survival models of microbiome-related parameters. These variables were age, sex, race, smoking status, histology, and chemotherapy (Supplementary Table 1).

### 16S rRNA gene sequencing

#### Assay

DNA was extracted with the DNeasy PowerLyzer PowerSoil DNA Isolation Kit (QIAGEN, Valencia, CA), following the manufacturer’s instructions. PCR amplification was performed on the 16S rRNA gene V4 hypervariable region using the 515F and 806R primers, with a 12-bp unique Golay barcoding [30]. PCR reactions were performed with an initial denaturation of 95 °C for 5 min, followed by 15 cycles of 95 °C for 1 min, 55 °C for 1 min, and 68 °C for 1 min, followed by 15 cycles of 95 °C for 1 min, 60 °C for 1 min, and 68 °C for 1 min, and a final extension for 10 min at 68 °C on a GeneAmp PCR System 9700 (Applied Biosystems, Foster City, CA). The PCR products were purified using a QIAquick Gel Extraction Kit (QIAGEN, Valencia, CA) and quantified using a Qubit 2.0 Fluorometric High Sensitivity dsDNA Assay (Life Technologies, Carlsbad, CA). PCR products were pooled relative to their band intensity. KAPA LTP Library Preparation Kit (KAPA Biosystems, Wilmington, MA) was used on the combined purified PCR products according to the manufacturer’s protocol and the size integrity of the amplicons with Illumina indices was validated with a 2100 Bioanalyzer (Agilent Technologies, Santa Clara, CA) at the Genomics Core at Albert Einstein College of Medicine. High-throughput amplicon sequencing was conducted on a HiSeq (Illumina, San Diego, CA) using 2 × 150 paired-end fragments.

#### Sequence read processing

Sequence reads were processed using QIIME 2 [31]. Briefly, sequence reads were demultiplexed, followed by quality filtering as described in Bokulich et al. [32]. Next, on the forward reads, the Deblur workflow was applied, which uses sequence error profiles to obtain putative error-free sequences, referred to as “sub” operational taxonomic units or amplicon sequence variants (ASVs) [33]. ASVs were assigned taxonomy using a naïve Bayes classifier pre-trained on the Greengenes [34] 13_8 99% OTUs, where the sequences have been trimmed to only include 150 bases from the 16S V4 region, bound by the 515F/806R primer pair. PICRUSt2 was used to predict abundance of functional pathways [35].

#### Quality control

Samples were processed in two batches, each including a negative PCR control and a positive PCR control (ZymoBIOMICS Microbial Community Standard, Zymo Research, Irvine, CA), and all sequenced together in a single run. Based on examination of the number of sequence reads in the tumor and normal lung samples, negative controls, and positive controls, we excluded samples with low sequence reads similar to the negative controls (<6000 reads/sample) (Supplementary Figure 2). After this exclusion, the median sequence read count per sample after the Deblur workflow was 54,864 (Q1= 33,598, Q3= 74,935). To remove potential contaminant sequences, we used two approaches as outlined in Cao et al. [36]: rare taxa filtering implemented using “PERFect” [37] and contaminant removal based on prevalence in negative controls and frequency among samples implemented using “decontam” [38]. Use of these complementary approaches in conjunction should remove contaminant taxa that are rare or abundant, respectively, and result in data with lower dimensionality, minimal information loss, reduced technical variability, and increased capacity for reproducibility and comparability across studies [36]. We performed contaminant filtering at each taxonomic level (i.e., phylum, class, order, etc.) separately, and then additionally removed contaminants at lower taxonomic levels that were identified as contaminants at higher taxonomic levels (e.g., removing taxonomic classes belonging to a contaminant phylum). After contaminant removal, the median sequence read count per sample was 16,418 (Q1=8,143, Q3=28,334). Contaminant-filtered data was used for all downstream processes, including running of PICRUSt2, calculation of α-diversity and β-diversity, and all statistical analyses described below.

The lowest sequencing depth among the samples, 2468 after contaminant removal, was sufficient to characterize the ranking of diversity among the samples, though not the ranking of number of observed ASVs (i.e., richness) (Supplementary Figure 3). Genus-level composition of the positive controls indicated appropriate detection of expected genera (Supplementary Figure 4). Among 4 samples run in duplicate, only 1 sample achieved sufficient sequencing depth in both duplicates (>2468 reads after contaminant removal); for this sample, compositional reproducibility was excellent, as was reproducibility for the two positive controls, based on similarity in principal coordinate analysis (Supplementary Figure 5). Additionally, we did not observe any compositional batch effect among the samples (Supplementary Figure 5).

### Quantitative PCR (qPCR)

A previously identified and published set of Clostridia specific primers (SJ-F/SJ-R) were selected [39]. Though cycling conditions were outlined in the initial publication, a range of annealing temperatures and cycling conditions were assessed to optimize performance. Assessments, similar to eventual qPCRs, were carried out on subsets of sample DNA, a ZymoBIOMICS Microbial Community DNA standard (D6305), an extraneous sample of stool DNA, and finally, reference *Clostridium difficile* gDNA, strain 4206, acquired from ATCC (BAA-1872D-5). A 2x Applied Biosystems SYBR Green PowerUp master mix (25742) was used and qPCR carried out on the Applied Biosystems Viia7 machine in the Albert Einstein College of Medicine Genomics core. Master mix was prepared according to the manufacturer’s instructions, primer concentrations in recommended range at 500 nM, with only deviation being the addition of MgCl_2_ to 0.5 uM. All samples were run in triplicate on a single Applied Biosystems 384-well plate (4309849). Lung sample DNA concentrations were measured, then subsequently normalized, and loaded to plate for a total of 15 ng DNA/well. qPCR was set to 40 amplification cycles with an annealing step of 15s at 53 °C and an extension of 60s at 72 °C. All other conditions followed master mix instructions. To generate a standard curve, seven dilutions of *C. difficile* gDNA were used, ranging from 10ng/well to 10fg/well.

### Gene expression

Gene expression was measured in buffy coat collected the day of and prior to tumor resection, using the nanoString® nCounter® PanCancer Immune Profiling Panel at the NYU Thoracic Oncology laboratory. Color-coded barcodes, attached to single target-specific probes corresponding to analytes of interest, hybridize directly to target molecules and are individually counted. The Panel provides multiplex gene expression analysis for 770 genes from 24 different immune cell types, common checkpoint inhibitors, and genes covering the adaptive and innate immune response. Gene expression data was normalized using housekeeping genes with the nSolver software.

### Statistical analysis

#### α-Diversity

α-Diversity (within-sample microbiome diversity) was assessed using richness (number of ASVs) and the Shannon diversity index, calculated using the QIIME 2 diversity plugin. The final values for each sample were calculated by averaging the richness and Shannon index from 100 iterations of rarefaction at 2468 sequence reads, the lowest sequence read depth among the samples after the exclusion of samples with low counts and contaminant filtering (described above).

#### β-Diversity

β-Diversity (between-sample microbiome diversity) was assessed using the Jensen-Shannon Divergence (JSD) [40]. Distances were calculated on the ASV level. Principal coordinate analysis (PCoA) [41] was used for visualization.

#### Comparison of the tumor and normal lung microbiome

Wilcoxon signed-rank tests were used to examine differences in α-diversity (number of ASVs, Shannon index) between paired tumor and normal lung samples. Permutational multivariate analysis of variance (PERMANOVA) [42] was used to examine whether overall bacterial composition (β-diversity) differed between paired tumor and normal lung samples, using patient ID as strata. PERMANOVA was also used to examine significant predictors of tumor and normal lung microbiome composition. To determine whether paired tumor-normal samples were more alike than unpaired samples, we used the JSD to calculate the average between-pair distance in overall bacterial composition for tumor and normal lung tissue sample pairs. We then calculated the average distances in 5000 permutations of random pairings of tumor and normal lung samples. If the mean pair distance was less than the 5th percentile of the permuted means, we concluded that the paired samples were more alike than random pairings of samples from different patients. For testing of differentially abundant taxa between tumor and normal lung samples, ASVs were agglomerated into phylum, class, order, family, genus, and species levels, in order to perform testing at each taxonomic level. Taxonomic abundance was transformed using the centered log-ratio (clr) transformation [43, 44] after adding a pseudocount, in order to remove compositional constraints of sequencing. Wilcoxon signed-rank tests were used to examine differences in taxonomic abundance between paired tumor and normal lung samples. *P*-values for taxa were adjusted for the false discovery rate (FDR) [45]; FDR adjustment was done at each taxonomic level (i.e., phylum, genus) separately.

#### Lung microbiome and survival

We used Cox proportional hazards models to determine whether α-diversity (number of ASVs, Shannon index) in the tumor or normal lung was associated with RFS, DFS, or OS, adjusting for age, sex, race, smoking status, histology, and chemotherapy. The community-level test of association between the microbiota and survival times (MiRKAT-S) [46] was used to test the association of overall bacterial composition (β-diversity), as measured by the JSD, in tumor or normal lung with RFS, DFS, and OS, adjusting for aforementioned covariates. Taxa and functional pathways associated with RFS, DFS, or OS were assessed independently in the tumor and normal lung samples, using repeated cross-validated elastic-net penalized Cox proportional hazards regression, as previously described [47]. For taxonomic analysis, ASVs were agglomerated into phylum, class, order, family, genus, and species levels, in order to perform testing at each taxonomic level. Taxonomic and functional pathway abundance was transformed using the centered log-ratio (clr) transformation [43, 44]. Agglomerated taxa missing taxonomic classification at the respective taxonomic level were removed (e.g., genera missing genus level classification), resulting in the inclusion of 6 phyla, 10 classes, 19 orders, 27 families, 41 genera, 13 species, and 787 ASVs. There were 359 functional pathways. We conducted 500 × 10-fold cross-validated elastic-net penalized Cox regression using the “cv.glmnet” function in the “glmnet” R package [48], with an *α* value of 0.5 to allow groups of correlated predictors to be selected together. Non-penalized covariates (age, sex, race, smoking status, histology, and chemotherapy) were included in each model. We summed the number of times each taxon or functional pathway was selected out of the 500 repetitions. For all tested taxa and functional pathways, we also fit standard Cox proportional hazards models for RFS, DFS, and OS, adjusting for the covariates listed above. *P*-values for these models were adjusted for the FDR [45]; FDR adjustment was done at each taxonomic level (i.e., phylum, genus) separately. We focused further on taxa and functional pathways selected ≥25% of the 500 times (125 times or more) and with FDR-adjusted *q* < 0.20. We used Spearman’s correlation to examine associations between the relative abundance of taxa and functional pathways.

#### Peripheral gene expression and survival

Gene expression data was log2 transformed for analysis. As shown above for microbiome, we used 500 × 10-fold cross-validated elastic-net penalized Cox regression to identify genes for which expression was related to RFS, DFS, or OS. We focused further on genes selected ≥25% of the 500 times (125 times or more) and with FDR-adjusted *q* < 0.20. The “topGO” package in R was used to determine gene ontology (GO) enrichments for survival-related genes. We used Spearman’s correlation to examine associations between survival-related genes and survival-related taxa and functional pathways.

#### Survival risk prediction

We considered whether the identified lung microbiome and/or peripheral gene expression biomarkers may improve risk prediction for RFS, DFS, or OS over standard covariates (age, sex, race, smoking status, histology, and chemotherapy) alone. Annual time-dependent area under the receiver operating characteristic (ROC) curve (AUC), starting at 1 year after surgical resection, were estimated using the R package “timeROC”, and 95% confidence intervals were constructed from the distribution of AUCs from 1000 bootstraps of the data. Permutation tests were used to test whether the biomarkers provide significant additional survival risk discrimination over standard covariates. Briefly, we generated null distributions of difference in time-dependent AUCs, by generating 5000 permutations of the microbiome and/or gene data while not disrupting the link of the survival time/censoring with the standard covariates. If the test statistic (difference in AUCs between microbiome and/or gene model and standard covariate model) was greater than the 95th percentile of the null distribution at a given time, we deemed that the biomarkers added significant (*p* < 0.05) risk discrimination over the standard covariates at that time.

### Validation cohorts

To validate observed findings of the current study, we included 16S microbiome sequencing data from two former studies: our previous pilot study of tumor and normal lung of NSCLC patients, referred to here as Peters et al. [21], and a previously described study of lower airway brushings of NSCLC patients, referred to here as Tsay et al. [17]. Sequencing data from Peters et al. [21] and Tsay et al. [17] was processed in QIIME2 as described above (see the “Sequence read processing” section). Contaminant filtering was performed at each taxonomic level using “PERFect” [37]. For Peters et al. [21], 16 tumor and 17 normal lung tissue samples were available, and covariates included in analysis were limited to age (years) and sex (male, female) due to low sample size. For Tsay et al. (2021), a total of 183 samples from 73 patients were available. Samples were categorized as “involved” (i.e., same side as tumor) or “non-involved”, and patients were categorized as having “local” (I-IIIA) or “advanced” (IIIB-IV) stage disease. Covariates included in the analysis were age (years), sex (male, female), race (white, non-white), smoking status (never, former, current), histology (adenocarcinoma, squamous cell carcinoma), chemotherapy (yes, no), and surgery (yes, no). To validate associations of selected taxa with survival, we fit Cox proportional hazards models for OS with clr-transformed taxa as predictors, adjusting for covariates (and accounting for within-patient clustering of samples in the case of Tsay et al.).

## Results

### Participant characteristics

Demographic and clinical characteristics of the 46 patients are presented in Table 1. The average patient age was 70 years old, and 50% were male, 87% were white, and 85% were former or current smokers. Half of the patients had lung adenocarcinomas (50%), and the other half had lung squamous cell carcinoma. Over a median of 4.8 years of follow-up (range 0.2–12.2 years), 43% and 9% experienced a recurrence or new primary cancer, respectively, and 50% died (Table 1). These characteristics were similar for patients with tumor samples (*n* = 39) and normal lung samples (*n* = 41) (Table 1).

**Table 1:** Characteristics of patients with stage II non-small cell lung cancer

	All	Patients with tumor sample	Patients with normal lung sample
*N*	46	39	41
Age, years, mean ± SD	70 ± 9	70.8 ± 8.7	70 ± 9.1
Male, *n* (%)	23 (50.0)	20 (51.3)	21 (51.2)
Race, *n* (%)			
White	40 (87)	35 (89.7)	37 (90.2)
Hispanic	4 (8.7)	2 (5.1)	4 (9.8)
Other	2 (4.3)	2 (5.1)	0 (0)
Smoking history, *n* (%)			
Never	7 (15.2)	6 (15.4)	4 (9.8)
Former	29 (63.0)	25 (64.1)	28 (68.3)
Current	10 (21.7)	8 (20.5)	9 (22.0)
Histology, *n* (%)			
Adenocarcinoma	23 (50.0)	20 (51.3)	18 (43.9)
Squamous cell carcinoma	23 (50.0)	19 (48.7)	23 (56.1)
Stage, *n* (%)			
IIA	30 (65.2)	26 (66.7)	26 (63.4)
IIB	16 (34.8)	13 (33.3)	15 (36.6)
Lymphovascular invasion, *n* (%)	24 (52.2)	21 (53.8)	21 (51.2)
Pleural invasion, *n* (%)	36 (78.3)	30 (76.9)	32 (78)
Tumor size, cm, mean ± SD	4.3 ± 2.1	4.3 ± 2.2	4.4 ± 2.1
Percent positive nodes, %, mean ± SD	13.0 ± 16.0	13.1 ± 17.0	13.4 ± 16.3
Chemotherapy, *n* (%)	15 (32.6)	13 (33.3)	12 (29.3)
Recurrence type, *n* (%)			
None	22 (47.8)	20 (51.3)	20 (48.8)
Nodal	6 (13.0)	5 (12.8)	6 (14.6)
Systemic	12 (26.1)	9 (23.1)	10 (24.4)
New primary	4 (8.7)	3 (7.7)	4 (9.8)
Multiple types	2 (4.3)	2 (5.1)	1 (2.4)
Alive at last follow-up, *n* (%)	23 (50.0)	19 (48.7)	21 (51.2)

### Characterization of microbiota in normal lung and tumor tissue

**Fig. 1:**
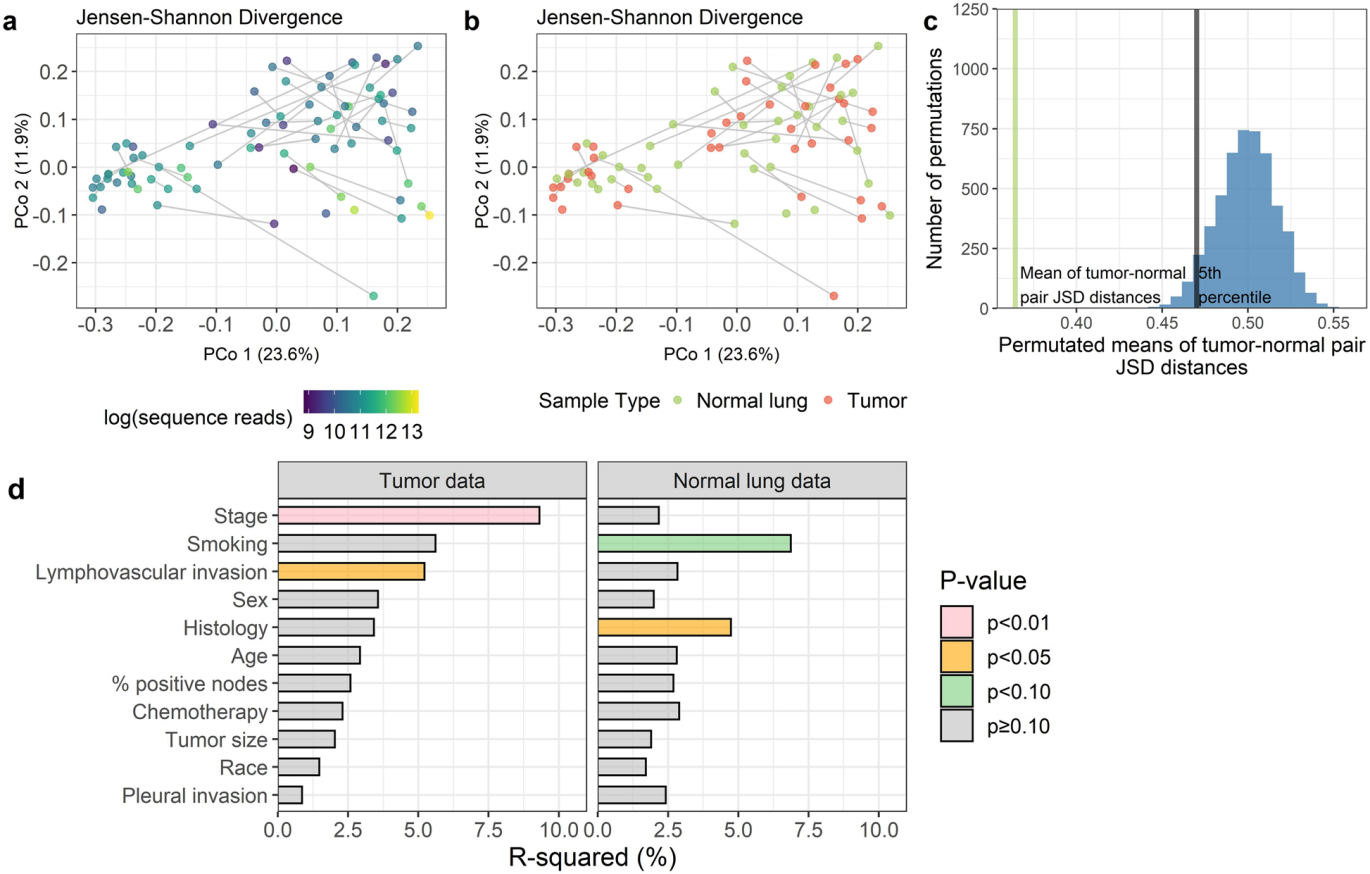
Determinants of tumor and normal lung sample composition in stage II NSCLC patients. **a**, **b** Principal coordinate analysis of the Jensen-Shannon Divergence, colored by **a** number of sequence reads or **b** tumor (*n* = 39) or normal lung (*n* = 41) sample type. Gray lines connect samples from the same patient. **c** Histogram of mean Jensen-Shannon Divergence for 5000 permutations of tumor vs. normal lung pairs; green line represents the observed mean distance for true tumor vs. normal lung pairs (*n* = 34) and black line represents the 5th percentile of the permuted distribution. **d** Association of patient characteristics with the Jensen-Shannon Divergence in tumor and normal lung, from PERMANOVA analysis

Sufficient sequencing depth for further analysis was obtained in 39 tumor and 41 normal lung samples. Paired tumor and normal lung samples from the same patient (*n* = 34 patients) did not differ significantly in the number of amplicon sequence variants (ASVs, i.e. richness, Wilcoxon signed-rank *p* = 0.65) or in the Shannon diversity index (*p* = 0.59) (Supplementary Figure 6). Paired tumor and normal lung samples also did not differ in overall microbiome composition, as measured by the Jensen-Shannon Divergence (JSD) (PERMANOVA *p* = 0.41). In fact, paired tumor and normal samples from the same patient were significantly more alike than permuted pairings of tumor and normal lung samples from different patients (*p* < 0.0001), and tumor and normal lung samples from the same patient tended to cluster together in principal coordinate analysis (Fig. 1a–c). Some patient clinical characteristics were related to overall microbiome composition: cancer stage and lymphovascular invasion were related to tumor microbiome composition, while cancer histology and smoking status were related to normal lung microbiome composition (all *p* < 0.10; Fig. 1d). The most common bacterial classes in the samples were *Gammaproteobacteria*, *Betaproteobacteria*, *Alphaproteobacteria*, *Bacilli*, *Clostridia*, and *Actinobacteria*, all present in 100% of samples, while the most abundant classes were *Actinobacteria* (mean [SD] relative abundance = 28.8% [18.1]), *Gammaproteobacteria* (mean [SD] = 23.9% [22.1]), and *Bacilli* (mean [SD] = 20.3% [17.8]) (Supplementary Figure 7). The most common genera in the samples were *Lactobacillus*, *Acinetobacter*, *Streptococcus*, *Corynebacterium*, and *Marmoricola*, all present in 100% of samples, while the most abundant genera were *Corynebacterium* (mean [SD] relative abundance = 16.9% [13.1]), *Marmoricola* (mean [SD] = 16.9% [22.0]), *Pseudomonas* (mean [SD] = 12.3% [17.6]), and *Acinetobacter* (mean [SD] = 10.1% [12.7]) (Supplementary Figure 8). We did not observe any differentially abundant taxa between paired tumor and normal lung samples (all *q* ≥ 0.17).

### Microbiome α- and β-diversity in relation to survival

Overall microbiome composition in the tumor samples, as measured by the JSD, was significantly related to DFS (*p* = 0.03), but not to RFS (*p* = 0.17) or OS (*p* = 0.45), while composition in normal lung samples was not related to RFS (*p* = 0.12), DFS (*p* = 0.45), or OS (*p* = 0.88), in MiRKAT-S tests adjusting for age, sex, race, histology, smoking status, and chemotherapy. Additionally, higher tumor microbiome Shannon diversity was associated with better DFS in a covariate-adjusted model (HR [95% CI] = 0.52 [0.28, 0.96], *p* = 0.04), but diversity of the normal lung microbiome was not associated with RFS, DFS, or OS (Supplementary Table 2).

### Microbial taxa related to survival

**Fig. 2:**
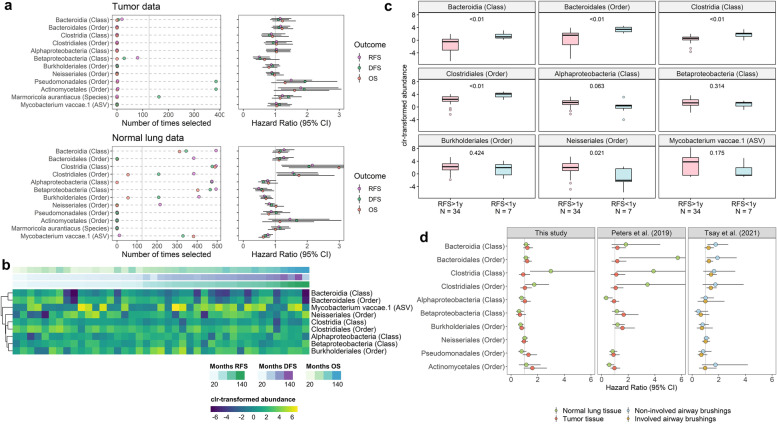
Microbial taxa related to survival in tumor and normal lung from stage II NSCLC patients. **a** For taxa selected >125 times in 500 × 10-fold cross-validated elastic-net penalized Cox regression and with FDR-adjusted *q* < 0.20 in either the tumor (*n* = 39) or normal lung (*n* = 41) data, we show number of times selected out of 500 times, and the hazard ratio (95% CI) from Cox proportional hazards regression of RFS, DFS, or OS on clr-transformed taxon abundance, adjusted for age, sex, race, histology, smoking status, and chemotherapy. **b** Heatmap of clr-transformed taxon abundance in normal lung tissue, sorted by months of RFS. **c** Boxplots of clr-transformed taxon abundance in normal lung tissue according to 1-year RFS status. *P*-values from Wilcoxon rank-sum test. **d** Comparison of study results with those from Peters et al. [21] and Tsay et al. [17]. Plots show the hazard ratio (95% CI) from Cox proportional hazards regression of OS on clr-transformed taxon abundance. Peters et al. (2019) included 16 tumor and 17 normal lung tissue samples, and models were adjusted for age and sex. Tsay et al. [17] included lower airway brushings from local stage (I-IIIA) NSCLC patients (*n* = 34 patients, *n* = 93 samples [36 involved, 57 non-involved]), and models were adjusted for age, sex, race, histology, smoking status, chemotherapy, and surgery; models also accounted for clustering of samples within patients

In 500 × 10-fold cross-validated elastic-net Cox regression models for RFS, DFS, and OS adjusting for covariates, we observed several taxa selected >25% of the time with *q* < 0.20 in the tumor and normal lung data (Fig. 2a; Supplementary Table 3); associations for the RFS, DFS, and OS outcomes tended to be similar in direction. In tumor tissue, higher abundance of orders Pseudomonadales and Actinomycetales, and species *Marmoricola aurantiacus*, was associated with worse survival, especially DFS (Fig. 2a). In normal lung tissue, higher abundance of classes Bacteroidia and Clostridia, and orders Bacteroidales and Clostridiales, was associated with worse survival, while higher abundance of classes Alphaproteobacteria and Betaproteobacteria, orders Burkholderiales and Neisseriales (from class Betaproteobacteria), and an ASV from *Mycobacterium vaccae*, was associated with better survival (Fig. 2a). Abundance of Bacteroidia/Bacteroidales and Clostridia/Clostridiales in normal lung appeared somewhat enriched in patients with short survival (Fig. 2b), and this was particularly the case for patients with < 1 year vs. > 1 year RFS (Fig. 2c), though this pattern was similar for 3-year and 5-year RFS (Supplementary Figure 9). In contrast, abundance of *Marmoricola aurantiacus* in tumor tissue was enriched only in patients with <1 year vs. >1 year RFS (Supplementary Figure 10).

To evaluate reproducibility, we examined whether any of the observed survival-related taxa were associated with overall survival in two validation cohorts: our previous pilot study of tumor and normal lung samples from NSCLC patients (Peters et al. [21]) and a study of lower airway brushings from NSCLC patients (Tsay et al. 2021) [17]. In Peters et al. [21], which included 17 stage I–IV NSCLC patients, we validated that Bacteroidales, Clostridia, and Clostridiales in normal lung (but not tumor) tissue were associated with worse survival (Fig. 2d). In 34 stage I–IIIA NSCLC patients from Tsay et al. [17], we validated that Bacteroidia and Bacteroidales in non-involved airway brushings, and Clostridia and Clostridiales in involved airway brushings, were associated with worse survival, though associations were similar for involved and non-involved samples (Fig. 2d). These associations were not observed in more advanced (stage IIIB–IV) NSCLC patients in Tsay et al. [17] (Supplementary Figure 11).

Since Clostridia abundance in normal lung tissue was associated with worse survival and validated in two validation cohorts, we performed qPCR to confirm the presence of Clostridia in the lung samples. The quantity of Clostridia in qPCR had a significant mild correlation with the clr-transformed abundance in the 16S data (Spearman *r* = 0.24, *p* = 0.03) (Supplementary Figure 12). Moreover, the qPCR-derived quantity of Clostridia in the normal lung tissue (log-transformed) was significantly associated with worse RFS, DFS, and OS in Cox regression models adjusting for covariates (HR [95% CI] = 2.5 [1.1, 5.5]; 2.4 [1.1, 5.2]; 3.2 [1.2, 8.3], respectively, and *p* = 0.02 for all), supporting our findings in the 16S data.

### Microbial functional pathways related to survival

**Fig. 3:**
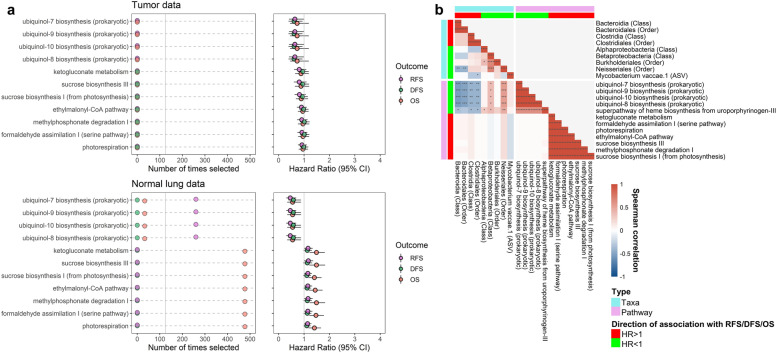
Microbial functional pathways related to survival in tumor and normal lung from stage II NSCLC patients. **a** For functional pathways selected >125 times in 500 × 10-fold cross-validated elastic-net penalized Cox regression and with FDR-adjusted *q* < 0.20 in either the tumor (*n* = 39) or normal lung (*n* = 41) data, we show number of times selected out of 500 times, and the hazard ratio (95% CI) from Cox proportional hazards regression of RFS, DFS, or OS on clr-transformed pathway abundance, adjusted for age, sex, race, histology, smoking status, and chemotherapy. **b** Spearman’s correlations between relative abundance of survival-related taxa and survival-related functional pathways in normal lung tissue data. *p<0.05, **p<0.01, ***p<0.001, ****p<0.0001

We observed five microbial functional pathways in the normal lung that were associated with better RFS, most related to ubiquinol biosynthesis, and 7 pathways in the normal lung associated with worse OS (Fig. 3a; Supplementary Table 4). No microbial functional pathways in the tumor data met our selection criteria for association with survival. The protective pathways in the normal lung were strongly positively correlated with each other, positively correlated with Betaproteobacteria and Neisseriales, and inversely correlated with Clostridia/Clostridiales and Bacteroidia/Bacteroidales in the normal lung (Fig. 3b). The 7 risk-associated pathways in the normal lung were strongly positively correlated with each other, but not correlated with any of the survival-related taxa in the normal lung (Fig. 3b). These risk-associated pathways belonged to various sugar biosynthesis and degradation classifications (Supplementary Table 5). We further examined which taxa may contribute to these functional pathways by conducting correlation analysis for all available taxa (phylum through ASV level), and found that taxa in the Neisseriales lineage were strongly related to the 5 protective pathways, while 3 ASVs from Methylobacteriaceae were strongly related to the 7 risk-associated pathways (Supplementary Figure 13). The Methylobacteriaceae ASVs were associated with worse OS (*p* < 0.05), but did not meet our selection criteria for significant association (Supplementary Table 3).

### Peripheral blood gene expression related to survival

**Fig. 4:**
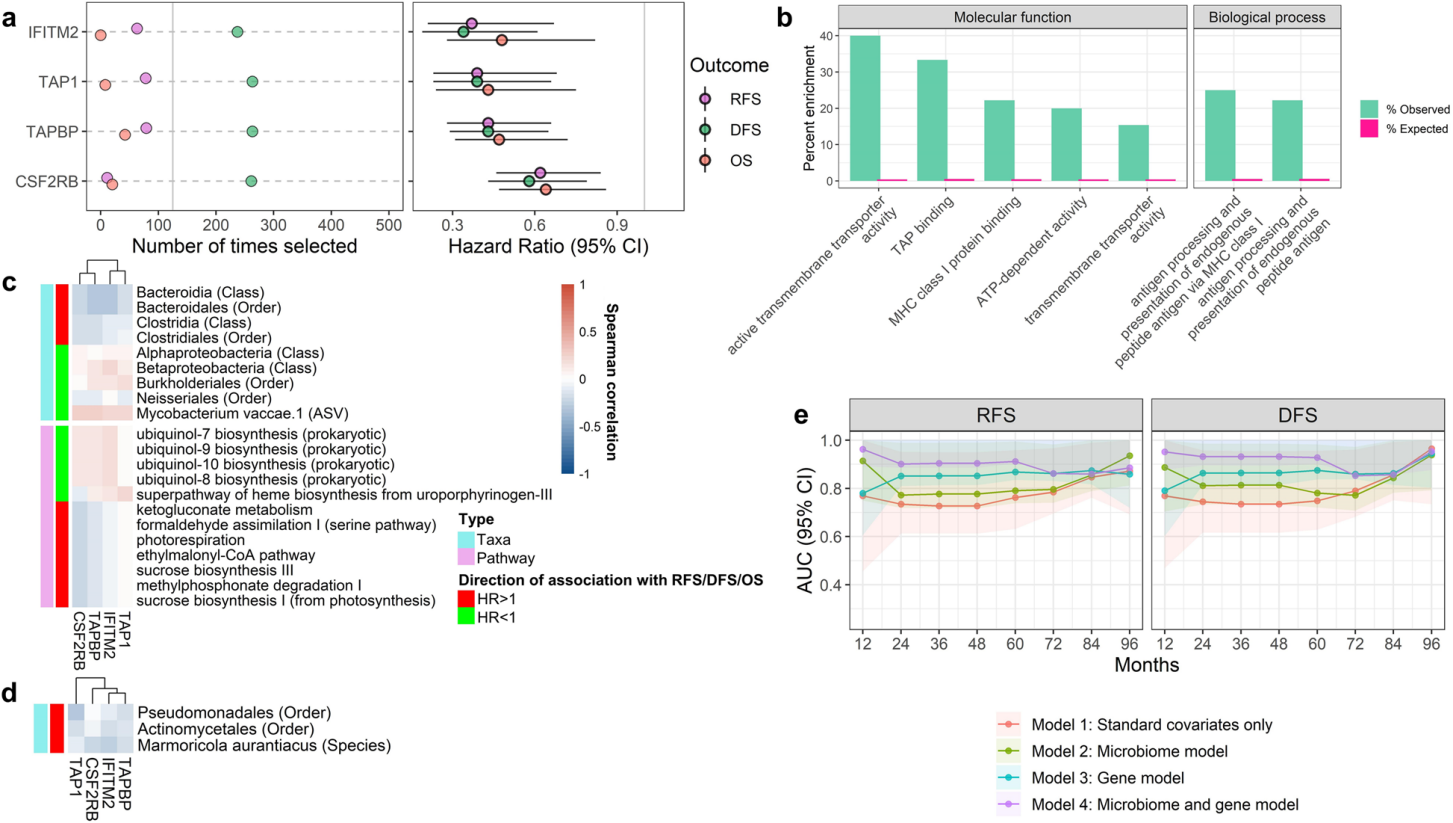
Peripheral blood gene expression related to survival in stage II NSCLC patients. **a** For genes selected >125 times in 500 × 10-fold cross-validated elastic-net penalized Cox regression and with FDR-adjusted *q* < 0.20, we show number of times selected out of 500 times, and the hazard ratio (95% CI) from Cox proportional hazards regression of RFS, DFS, or OS on log2-transformed gene expression, adjusted for age, sex, race, histology, smoking status, and chemotherapy. **b** Gene ontology (GO) enrichment for survival-related genes. GO terms from the biological process or molecular function ontologies with *p* < 0.001 from Fisher’s exact test are shown. **c**, **d** Partial Spearman’s correlations between relative abundance of survival-related taxa and functional pathways in **c** normal lung or **d** tumor and survival-related gene expression. Correlations were adjusted for age, sex, race, histology, smoking status, and chemotherapy. *p<0.05, **p<0.01, ***p<0.001, ****p<0.0001. **e** Area under the time-dependent ROC curve (AUC) for the four models at different follow-up times. Model 1 included standard covariates only (age, sex, race, histology, smoking status, and chemotherapy); model 2 (microbiome model) included standard covariates and clr-transformed Clostridiales and Bacteroidales abundance in the normal lung; model 3 (gene model) included standard covariates and log2-transformed peripheral expression of IFITM2, TAP1, TAPBP, and CSF2RB; and model 4 (microbiome and gene model) included standard covariates plus the microbiome and gene variables. Solid lines (circles) represent the time-dependent AUCs from “timeROC” R package; shaded area represents the 95% confidence intervals determined from 1000 bootstraps

We observed 4 genes for which expression was associated with better survival: IFITM2, TAP1, TAPBP, and CSF2RB (Fig. 4a; Supplementary Table 6). This set of genes were enriched (*p* < 0.001) for functions of transmembrane transporter activity and antigen processing and presentation of peptides via MHC Class I (Fig. 4b); these enrichments were solely related to the TAP1 and TAPBP genes. The survival-related genes were not significantly associated with any survival-related taxa or functional pathways in the normal lung (Fig. 4c) or tumor (Fig. 4d), in partial correlations adjusted for age, sex, race, histology, smoking status, and chemotherapy.

### Survival risk prediction models

We examined whether lung microbiome and peripheral gene expression biomarkers could improve survival risk prediction over standard risk factors alone, in a subset of 37 participants with both normal lung microbiome and gene expression data available. Clostridiales and Bacteroidales in the normal lung were included as microbiome biomarkers, given their reproducibility in the two validation cohorts, and IFITM2, TAP1, TAPBP, and CSF2RB were included as peripheral gene expression biomarkers. We evaluated the performance of four models for risk prediction of RFS and DFS: (1) a model with standard covariates (age, sex, race, histology, smoking status, and chemotherapy); (2) a model with standard covariates plus clr-transformed Clostridiales and Bacteroidales abundance in the normal lung (microbiome model); (3) a model with standard covariates plus log2-transformed IFITM2, TAP1, TAPBP, and CSF2RB expression in peripheral blood (gene model); and (4) a model with standard covariates plus the microbiome and gene biomarkers (microbiome and gene model). The microbiome model had a higher AUC than the standard covariate model at 1 year of follow-up for RFS and DFS, after which it performed marginally better than the standard covariate model at years 2–4 of follow-up for DFS, but not RFS (Fig. 4e; Supplementary Figures 14 and 15). In contrast, the gene model performed significantly better than the standard covariate model at years 2–5 (but not year 1) of follow-up for RFS and DFS (Fig. 4e; Supplementary Figures 14 and 15). Consequently, the microbiome and gene biomarkers together performed significantly better than the standard covariate model at years 1–5 of follow-up for RFS and DFS (Fig. 4e; Supplementary Figures 14 and 15). Similarly, microbiome biomarkers added significant risk discrimination over standard covariates and gene biomarkers at year 1 of follow-up for RFS and years 1–4 for DFS, while gene biomarkers added significant risk discrimination over standard covariates and microbiome biomarkers at years 2–5 of follow-up for RFS and DFS (Supplementary Figures 14 and 15).

## Discussion

In this study of stage II NSCLC patients, we observed that taxa in the tumor and normal lung microbiome, and the expression of 4 genes in peripheral blood, were predictive of lung cancer recurrence risk. Notably, the associations of Clostridia/Clostridiales and Bacteroidia/Bacteroidales in the normal lung with worse survival were validated in two independent studies. Together, lung microbiome and peripheral gene expression biomarkers provided significant additional risk discrimination for recurrence risk over standard demographic and clinical information during the first several years after tumor resection, with microbiome biomarkers performing better for short-term (~1 year) prediction and gene expression biomarkers performing better for longer-term (2–5 years) prediction. Our results suggest that biomarkers in normal lung tissue and peripheral blood could improve recurrence risk prediction in NSCLC.

A few previous studies have examined the relationship of the lung microbiome with the risk of lung cancer recurrence. In 48 early-stage NSCLC patients, Patnaik et al. observed a microbiome signature in pre-surgery bronchoalveolar lavage samples, based on 19 genera, that was strongly associated with recurrence risk and tumor gene expression [18]. They also found three genera in tumor tissue (*Staphylococcus*, *Bacillus*, and *Anaerobacillus*) that were related to recurrence risk, but no associations were found for the adjacent non-tumor lung tissue microbiome [18], in contrast with our results. In 83 lung cancer patients (38 with early-stage NSCLC), Tsay et al. reported that a lower airway microbiome similar to that of the oral cavity (i.e., enriched in taxa such as *Streptococcus, Prevotella*, and *Veillonella)* was associated with poorer survival in stage I–IIIA NSCLC, and that this oral-enriched signature was associated with upregulation of p53, PI3K/PTEN, ERK, and IL6/IL8 pathway transcription in the lower airway [17]. We re-analyzed data from this study to find that higher abundance of Clostridia and Bacteroidia in lower airway brushings was associated with worse survival for stage I-IIIA NSCLC patients. This independent validation supports that the relationship of these taxa with patient outcome is less likely to be a chance finding, and may even reflect involvement of these lung bacteria in the mechanism of NSCLC recurrence.

Given that bacterial classes and orders, such as those associated with survival in our study (Clostridia/Clostridiales, Bacteroidia/Bacteroidales, Alphaproteobacteria, and Betaproteobacteria/Burkholderiales/Neisseriales in the normal lung; Actinomycetales and Pseudomonadales in tumor), are broad groups featuring many constituent species, it is difficult to surmise underlying mechanisms for their association with recurrence. Alphaproteobacteria, Betaproteobacteria, and Pseudomonadales, belonging to phylum Proteobacteria, and Bacteroidia, from phylum Bacteroidetes, are all gram-negative bacteria, meaning they possess an outer lipopolysaccharide (LPS) membrane, while Clostridia, from phylum Firmicutes, and Actinomycetales, from phylum Actinobacteria, are generally gram-positive bacteria which lack this outer membrane. LPS is known for its potent activation of the innate immune system through its binding to toll-like receptor 4 (TLR4) [49], important for pathogen clearance [50]. However, there are many nuances in LPS structure across species, such that LPS from Proteobacteria is considered immunostimulatory, while LPS from Bacteroidetes is considered non-stimulatory and possibly antagonistic to TLR4 [50]. Proteobacteria (mostly Gammaproteobacteria) are enriched in the lungs in a variety of inflammatory lung diseases, including asthma and chronic obstructive pulmonary disease [15]. Additionally, gram-negative bacteria have been shown to increase metastasis via TLR4 activation in animal models of NSCLC [51], and NSCLC patients with gram-negative bacterial pulmonary infection [52] or tumor TLR4 overexpression [53] have shorter time to recurrence. Thus, the association of Pseudomonadales (from class Gammaproteobacteria) in tumor tissue with worse survival was in line with prior evidence, but it was surprising that Alphaproteobacteria and Betaproteobacteria in the normal lung were related to improved survival in our study. It is possible that non-pathogenic lung Proteobacteria could also play a role in anti-cancer immune surveillance. In support of this, a study investigating aerosol antibiotic therapy for lung metastases in mice found that treatment with aerosol antibiotics shifted the lung microbiome composition in favor of increased Proteobacteria and decreased Firmicutes, which was associated with an enhanced immune response against cancer cells and reduced number of lung metastases [54]. However, our findings for Alphaproteobacteria, Betaproteobacteria, and Pseudomonadales were not replicated in our two validation cohorts; thus, validity of these associations is unclear. On the other hand, the associations of Clostridia/Clostridiales and Bacteroidia/Bacteroidales in the normal lung with worse survival were replicated in the two validation cohorts. These taxa have been identified as common residents of the lung microbiome in other studies [55–57], though their putative function in the lung microbiome community is unknown.

At lower taxonomic levels, we observed that species *Marmoricola aurantiacus* in tumor tissue was associated with worse DFS only, and an ASV from *Mycobacterium vaccae* in normal lung tissue was related to better survival. *Mycobacterium* [55, 56] and *Marmoricola* [56, 58] have been previously observed in the respiratory microbiome in other studies, but not consistently across all studies, making it unclear if these are common residents of the lung microbiome or possible contaminants. As *Marmoricola* was an abundant genus in our samples, its relation with worse DFS may explain the significant association of overall tumor microbiome composition with DFS in MiRKAT-S analysis. Heat-killed *Mycobacterium vaccae* injection has been shown to improve survival in later-stage NSCLC when used as an adjuvant therapy [59], which is related to its immune regulation role [60] and in line with the observed protective association.

Analysis of inferred functional capacity in the lung microbiome revealed ubiquinol biosynthesis pathways in the normal lung as protective for lung cancer recurrence, while sugar synthesis and metabolism pathways in the normal lung were related to worse survival. The very high correlations within the groups of protective and risk-associated pathways, respectively, suggest that their associations with survival are driven by specific taxa which possess these functions. Indeed, the protective and risk-associated pathways appeared to stem from Neisseriales and Methylobacteriaceae ASVs, respectively. Thus, it is not clear whether these pathways may have causal involvement in lung cancer recurrence or are simply reflective of survival-related taxa. However, ubiquinol (also known as coenzyme Q10) is a naturally occurring antioxidant and its levels in plasma have been associated with reduced lung cancer risk in a prospective study [61], consistent with a protective role of microbial ubiquinol biosynthesis.

We did not observe a significant difference in overall microbiome composition between paired tumor and normal lung samples in our study, with composition being more similar within than between patients. Similar overall composition for paired tumor and normal lung is consistent with our previous pilot study [21] and other studies that have compared tumor and normal lung samples [18, 55, 56, 62–65]. Some of these studies have observed differences in specific taxa between paired tumor and normal lung samples, including a meta-analysis of 5 studies which found decreased abundance of Actinobacteria, *Corynebacterium*, *Lachnoanaerobaculum*, and *Halomonas* in tumor compared to adjacent normal lung [66]. While we may expect that changes in the lung due to the tumor would lead to microbiome dysbiosis in tumor compared to adjacent normal lung, a few factors may contribute to the lack of findings in this regard. First, smaller sample size, such as in this study, may reduce the power to detect differences in composition and specific taxa. Second, tumor and normal lung samples may differ within a patient, but not in a systematic way across all patients (i.e. each tumor-normal pair may be unique in its differences). Third, earlier stage NSCLC may have less impact on the tumor microbiome than more advanced disease. Lastly, it is possible that normal lung samples are still too proximal to tumors for differentiation, and more distant normal samples are necessary to observe the influence of the tumor on the microbiome. Despite observed similarities in tumor and normal lung within patients, different associations of the microbiome with survival were observed for tumor and normal lung. This could be due to truly unique associations for each sample type with survival, or it could be due to the different sets of patients with tumor and normal lung data in this study (only 34 patients had both sample types). This is plausible since we observed some consistent associations for both tumor and normal lung (e.g., Betaproteobacteria with better survival) which were more robust in the normal lung data, and therefore only selected as significant in the penalized regressions for normal lung. Interestingly, cancer stage (IIA or IIB) was significantly associated with overall composition of the tumor (but not normal lung) microbiome, suggesting that the progressing tumor microenvironment does influence the tumor microbiota.

We also evaluated the relationship of peripheral blood immune gene expression with recurrence, and observed 4 genes (TAP1, TAPBP, CSF2RB, and IFITM2) that were associated with better survival. These genes were not among those prognostic for survival in a previous study of PBMC gene expression based on microarray in 108 stage I–IIIA NSCLC patients. In that study, a gene prognostic score of 26 genes was identified in a training set of 54 patients using penalized Cox proportional hazards regression and validated in the other set of 54 patients [28]. They found a total of 1704 genes associated with survival at *p* < 0.05, but the list of genes was not provided; genes associated with better survival were enriched for functions related to ribosomal structure and function, while genes associated with worse survival were enriched for cell cycle or metaphase functions [28]. In regard to our findings, Transporter Associated With Antigen Processing (TAP1) and TAP Binding Protein (TAPBP) are members of the peptide loading complex which facilitates antigen presentation on MHC class I molecules [67]. MHC class I presentation of antigens on cancer cells triggers recognition by cytotoxic T-cells and destruction of the cancer cell, making TAP1 and TAPBP critical for anti-cancer immunosurveillance [68]. While loss of MHC class I expression in NSCLC tumors is a mechanism of immune escape and a significant predictor of poor prognosis [68], this has not been studied for peripheral blood. However, MHC class I expression in peripheral blood may reflect tumor expression, since PBMC expression in cancer patients is significantly lower than healthy controls for several cancer types [69–71]. This may explain the association of TAP1 and TAPBP expression in peripheral blood with better survival in our study. Colony-stimulating factor 2 receptor subunit beta (CSF2RB) is a subunit of the receptor for interleukin-3, interleukin-5, and granulocyte-macrophage colony-stimulating factor (GM-CSF). GM-CSF has been controversially related to both pro-cancer and anti-cancer functions, but its utilization as an immune adjuvant in cancer immunotherapy clinical trials [72] suggests that it can enhance antitumor efficacy. Higher CSF2RB expression in lung adenocarcinoma tumors is associated with better survival [73], in line with our observations in peripheral blood. Lastly, Interferon-Induced Transmembrane Protein 2 (IFITM2) is a member of a protein family which inhibits viral entry into host cells [74]. Though IFITM2 has not been previously studied in lung cancer prognosis, the related IFITM1 was associated with poor survival in lung adenocarcinoma, contrary to the protective effect of IFITM2 observed in our study.

Survival-related lung microbiota and survival-related peripheral gene expression were uncorrelated with each other, suggesting there are independent mechanisms for their respective relationships with survival (i.e., the lung microbiota may act more locally). Analysis of annual time-dependent AUCs revealed that lung microbiome biomarkers were only useful in short-term (1-year) prediction of recurrence, significantly improving the AUC from the standard covariate model. In contrast, peripheral blood gene expression biomarkers significantly improved the AUC for longer-term (2–5-year) prediction of recurrence. This may indicate that each data type provides unique information regarding time to recurrence. Together, the lung microbiome and peripheral blood gene expression biomarkers significantly improved recurrence risk prediction during years 1–5 after resection, to a greater extent than either set of biomarkers alone. While the joint utility of these biomarkers will need to be validated in a larger study, these preliminary findings suggest that novel biomarkers from the lung microbiome and peripheral blood may hold promise for predicting prognosis in early-stage NSCLC.

## Conclusions

In summary, we show that Clostridia and Bacteroidia in normal lung tissue may be reproducible biomarkers of recurrence risk after tumor resection in early-stage NSCLC patients. Additionally, expression of TAP1, TAPBP, CSF2RB, and IFITM2 in peripheral blood were associated with better survival, and the combination of lung microbiome and peripheral gene expression biomarkers significantly improved prediction of recurrence in years 1–5 after resection, over standard covariates alone. These findings will require validation in other larger study populations. This study faced several limitations that future studies can address, including the small sample size, limited sample types (e.g., only stage II, no healthy controls), lack of shotgun metagenomic sequencing to determine actual rather than inferred functional potential of the lung microbiome, and lack of host gene expression data in lung tissue which would be more proximal to the lung microbiota. However, we identified compelling biomarkers in under-explored data types—the lung microbiome and peripheral blood gene expression—which may improve risk prediction of recurrence in NSCLC patients and thus improve survival if implemented in a clinical setting. In the future, these biomarkers can also be tested for their predictive ability in the response to chemotherapy or immunotherapy (we did not have a sufficient sample size of patients receiving chemotherapy to evaluate response to therapy in this study). The biomarkers may also suggest targets for experimental evaluation in animal models of the role of the lung microbiota or peripheral immunity in lung cancer recurrence. Better understanding of the mechanistic role of the lung microbiota and peripheral immunity may lead to the development of new adjuvant therapies for NSCLC, which may improve lung microbiome and systemic immune profiles to prevent cancer recurrence.

## Supplementary Information

**Additional file 1: Supplementary Table 1.** Predictors of RFS, DFS, and OS in Cox proportional hazards models. Adjusted models are adjusted for all other covariates shown here. **Supplementary Table 2.** Association of richness and the Shannon diversity index with RFS, DFS, and OS in Cox proportional hazards models. Analyses were performed on the tumor tissue microbiome data and normal tissue microbiome data. Adjusted models are adjusted for age, sex, race, histology, smoking, and chemotherapy. **Supplementary Table 3.** Association of taxa with RFS, DFS, and OS in Cox proportional hazards models. Analyses were performed on the tumor tissue microbiome data and normal tissue microbiome data. All models are adjusted for age, sex, race, histology, smoking, and chemotherapy. We also performed 500 × 10-fold cross-validated elastic-net penalized Cox regression, and show the number of times each taxa was selected out of 500. Penalized regression and FDR-correction were performed on each taxonomic level separately. **Supplementary Table 4.** Association of functional pathways with RFS, DFS, and OS in Cox proportional hazards models. Analyses were performed on the tumor tissue microbiome data and normal tissue microbiome data. All models are adjusted for age, sex, race, histology, smoking, and chemotherapy. We also performed 500 × 10-fold cross-validated elastic-net penalized Cox regression, and show the number of times each function was selected out of 500. **Supplementary Table 5.** Information on MetaCyc pathways related to RFS, DFS, or OS. **Supplementary Table 6.** Association of gene expression in buffy coat with RFS, DFS, and OS in Cox proportional hazards models. All models are adjusted for age, sex, race, histology, and smoking. We also performed 500 × 10-fold cross-validated elastic-net penalized Cox regression, and show the number of times each gene was selected out of 500. Genes highlighted in yellow are those correlated (*p*<0.01) with at least one survival-related taxa/functional pathway in normal lung.

**Additional file 2: Supplementary Figure 1.** Flow diagram of participants and samples included in the study. **Supplementary Figure 2.** (a) 16S band intensity relative to background, in gels of sample PCR products, by batch. (b) Number of sequence reads per sample after the Deblur workflow, by batch. (c) Number of sequence reads per sample after contaminant removal, by batch. (d) Scatterplot of relative band intensity vs. number of sequence reads per sample after the Deblur workflow. (e) Scatterplot of relative band intensity vs. number of sequence reads per sample after contaminant removal. **Supplementary Figure 3.** Rarefaction curves of (a) the number of observed ASVs and (b) the Shannon diversity index. At each depth, the average number of ASVs or Shannon index over 100 iterations was calculated. Vertical dotted line at 2,468 sequence reads represents the lowest depth among the samples after the Deblur workflow, removal of contaminants, and exclusion of samples with low read counts. **Supplementary Figure 4.** (a) Relative abundance of genera in the two positive controls (ZymoBIOMICS Microbial Community Standard, Zymo Research, Irvine, CA). Only genera with relative abundance >0.1% are shown. (b) Scatter plot of the relative abundance of these genera in the first and second positive control. The red dot signifies the expected abundance (12%) of the following species: *Listeria monocytogenes, Pseudomonas aeruginosa, Bacillus subtilis, Escherichia coli, Salmonella enterica, Lactobacillus fermentum, Enterococcus faecalis, Staphylococcus aureus*. **Supplementary Figure 5.** Principal coordinate analysis of (a-c) the generalized UniFrac distance and (d-f) the Jensen-Shannon Divergence. From left to right, plots are colored by sample type, batch, and the number of sequence reads after Deblur and contaminant removal. In the right-most plots, lines connect duplicate samples. **Supplementary Figure 6.** Boxplots of (a) the number of observed ASVs and (b) the Shannon diversity index in tumor and normal lung samples at the lowest sequencing depth (2,468 sequence reads) after the Deblur workflow, contaminant removal, and exclusion of samples with low read counts. Lines connect samples from the same patient. **Supplementary Figure 7.** (a) Frequency of common classes in lung samples. Y-axis represents the number of samples (out of 80 samples total). Plot shows all classes present in >50% of the lung samples. (b) Relative abundance of classes in lung samples. Plot shows all classes with mean relative abundance >1%. **Supplementary Figure 8.** (a) Frequency of common genera in lung samples. Y-axis represents the number of samples (out of 80 samples total). Plot shows all genera present in >50% of the lung samples. (b) Relative abundance of genera in lung samples. Plot shows all genera with mean relative abundance >1%. **Supplementary Figure 9.** Boxplots of clr-transformed taxon abundance in normal lung tissue according to 1-year, 3-year, 5-year, and 7-year RFS status. P-values from Wilcoxon rank-sum test. **Supplementary Figure 10.** (a) Heatmap of clr-transformed taxon abundance in normal lung tissue, sorted by months of RFS. (b) Boxplots of clr-transformed taxon abundance in tumor tissue according to 1-year, 3-year, 5-year, and 7-year RFS status. P-values from Wilcoxon rank-sum test. **Supplementary Figure 11.** Plots show the hazard ratio (95% CI) from Cox proportional hazards regression of OS on clr-transformed taxon abundance. Tsay et al. (2021) included lower airway brushings from local stage (I-IIIA) (n=34 patients, n=93 samples [36 involved, 57 non-involved]), and advanced stage (IIIB-IV) (n=39 patients, n=90 samples [40 involved, 50 non-involved]) NSCLC patients. Models were adjusted for age, sex, race, histology, smoking status, and chemotherapy (and surgery in local stage); models also accounted for clustering of samples within patients. **Supplementary Figure 12.** Scatter plot of clr-transformed Clostridia abundance (from 16S data) with qPCR quantity of Clostridia (log-transformed axis). A pseudocount (half the minimum non-zero quantity) was added to the qPCR quantity to allow log transformation. Spearman correlation and p-value are shown on the plot, along with the linear regression line. **Supplementary Figure 13.** Spearman’s correlations of taxa associated with survival-related functional pathways in normal lung. Relative abundance of taxa and pathways was used in analysis. Only taxa with correlations ≥0.5 are included in the heatmap. *p<0.05, **p<0.01, ***p<0.001, ****p<0.0001. **Supplementary Figure 14.** Null distributions of difference in time-dependent AUC for RFS, from 5000 permutations. Solid black line represents the observed (non-permuted) difference in AUC; dotted line represents the 95th percentile of the permuted distribution. **Supplementary Figure 15.** Null distributions of difference in time-dependent AUC for DFS, from 5000 permutations. Solid black line represents the observed (non-permuted) difference in AUC; dotted line represents the 95th percentile of the permuted distribution.

## Data Availability

The 16S rRNA sequencing data and patient demographic and clinical information are available in the Sequence Read Archive repository, under BioProject accession number PRJNA773392 (https://www.ncbi.nlm.nih.gov/bioproject/773392) [75].

## Abbreviations

ASV: Amplicon sequence variant
AUC: Area under the receiver operating characteristic curve
clr: Centered log ratio
DFS: Disease-free survival
HR: Hazard ratio
JSD: Jensen-Shannon Divergence
NSCLC: Non-small cell lung cancer
OS: Overall survival
PERMANOVA: Permutational multivariate analysis of variance
RFS: Recurrence-free survival
ROC: Receiver operating characteristic
