# Supplementary material for: The lung microbiome, peripheral gene expression, and recurrence-free survival after resection of stage II non-small cell lung cancer
Source: Genome Med. 2022 Oct 27;14:121. doi: 10.1186/s13073-022-01126-7 (PMC9609265; doi:10.1186/s13073-022-01126-7)
Supplement: Supplementary file 2 — Additional file 2: Supplementary Figure 1. Flow diagram of participants and samples included in the study. Supplementary Figure 2. (a) 16S band intensity relative to background, in gels of sample PCR products, by batch. (b) Number of sequence reads per sample after the Deblur workflow, by batch. (c) Number of sequence reads per sample after contaminant removal, by batch. (d) Scatterplot of relative band intensity vs. number of sequence reads per sample after the Deblur workflow. (e) Scatterplot of relative band intensity vs. number of sequence reads per sample after contaminant removal. Supplementary Figure 3. Rarefaction curves of (a) the number of observed ASVs and (b) the Shannon diversity index. At each depth, the average number of ASVs or Shannon index over 100 iterations was calculated. Vertical dotted line at 2,468 sequence reads represents the lowest depth among the samples after the Deblur workflow, removal of contaminants, and exclusion of samples with low read counts. Supplementary Figure 4. (a) Relative abundance of genera in the two positive controls (ZymoBIOMICS Microbial Community Standard, Zymo Research, Irvine, CA). Only genera with relative abundance >0.1% are shown. (b) Scatter plot of the relative abundance of these genera in the first and second positive control. The red dot signifies the expected abundance (12%) of the following species: Listeria monocytogenes, Pseudomonas aeruginosa, Bacillus subtilis, Escherichia coli, Salmonella enterica, Lactobacillus fermentum, Enterococcus faecalis, Staphylococcus aureus. Supplementary Figure 5. Principal coordinate analysis of (a-c) the generalized UniFrac distance and (d-f) the Jensen-Shannon Divergence. From left to right, plots are colored by sample type, batch, and the number of sequence reads after Deblur and contaminant removal. In the right-most plots, lines connect duplicate samples. Supplementary Figure 6. Boxplots of (a) the number of observed ASVs and (b) the Shannon diversity index in tumor a [file 13073_2022_1126_MOESM2_ESM.docx]

**SUPPLEMENTARY MATERIAL**


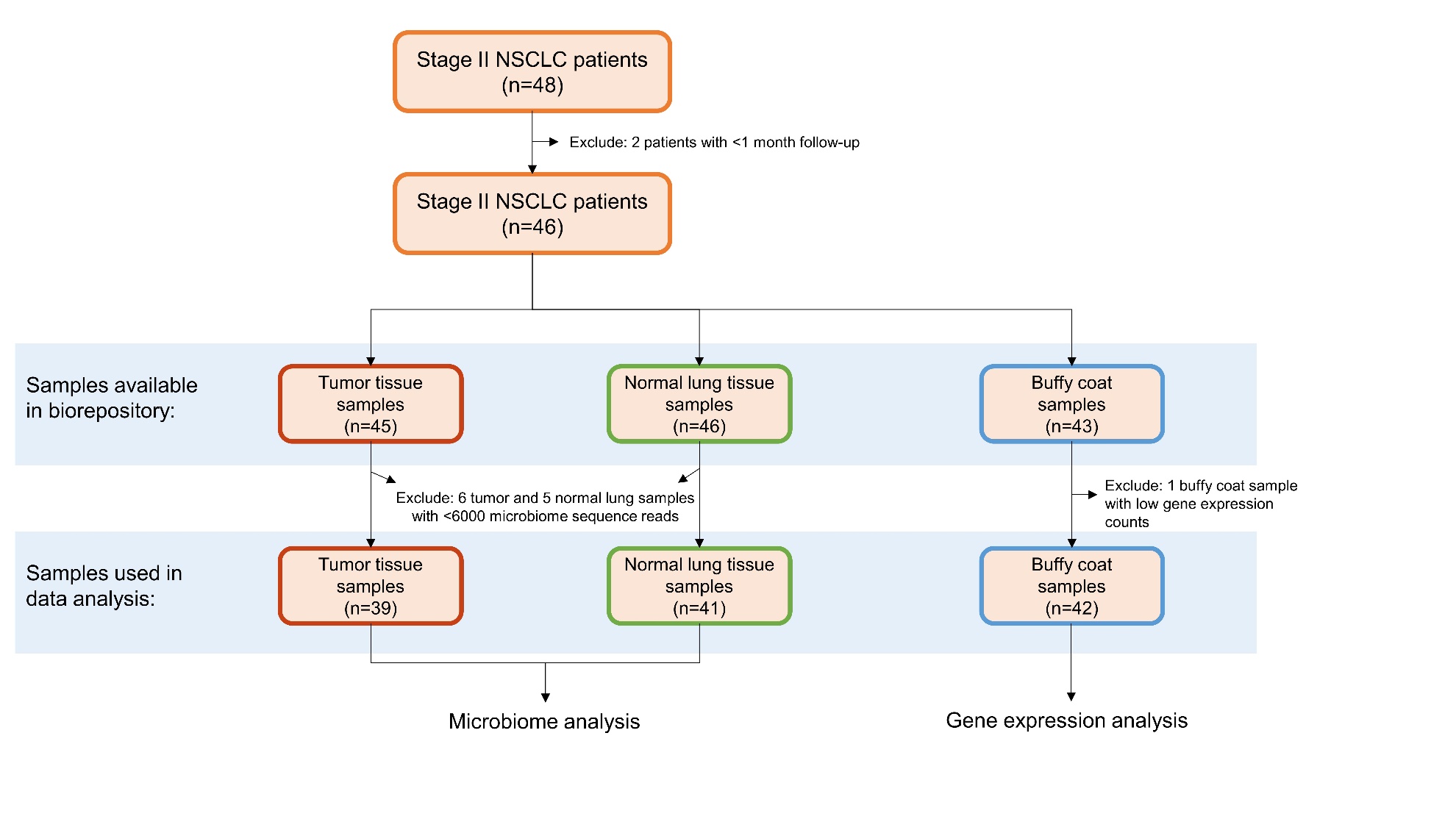


**Supplementary Figure 1.** Flow diagram of participants and samples included in the study.


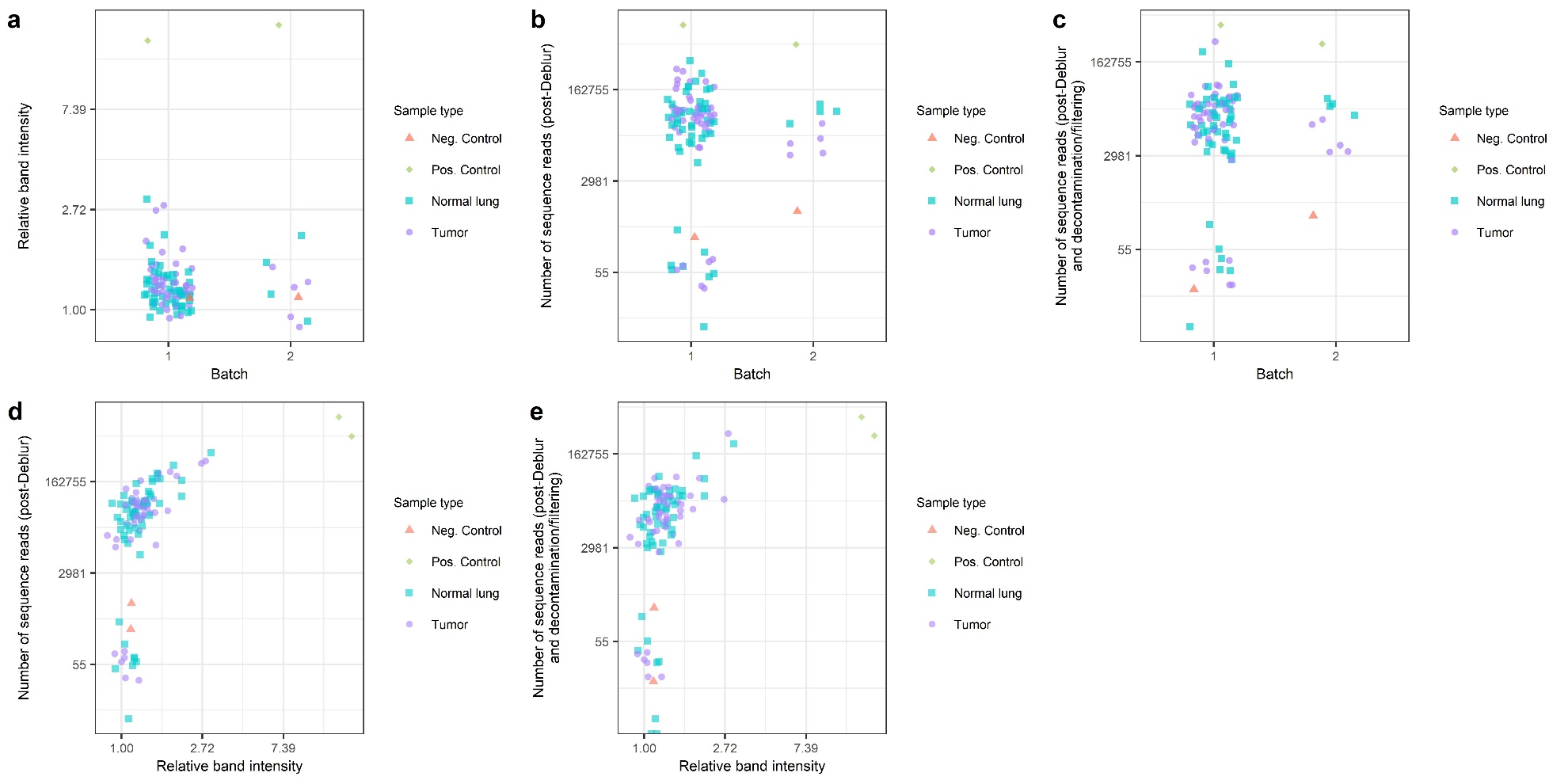


**Supplementary Figure 2.** (a) 16S band intensity relative to background, in gels of sample PCR products, by batch. (b) Number of sequence reads per sample after the Deblur workflow, by batch. (c) Number of sequence reads per sample after contaminant removal, by batch. (d) Scatterplot of relative band intensity vs. number of sequence reads per sample after the Deblur workflow. (e) Scatterplot of relative band intensity vs. number of sequence reads per sample after contaminant removal.


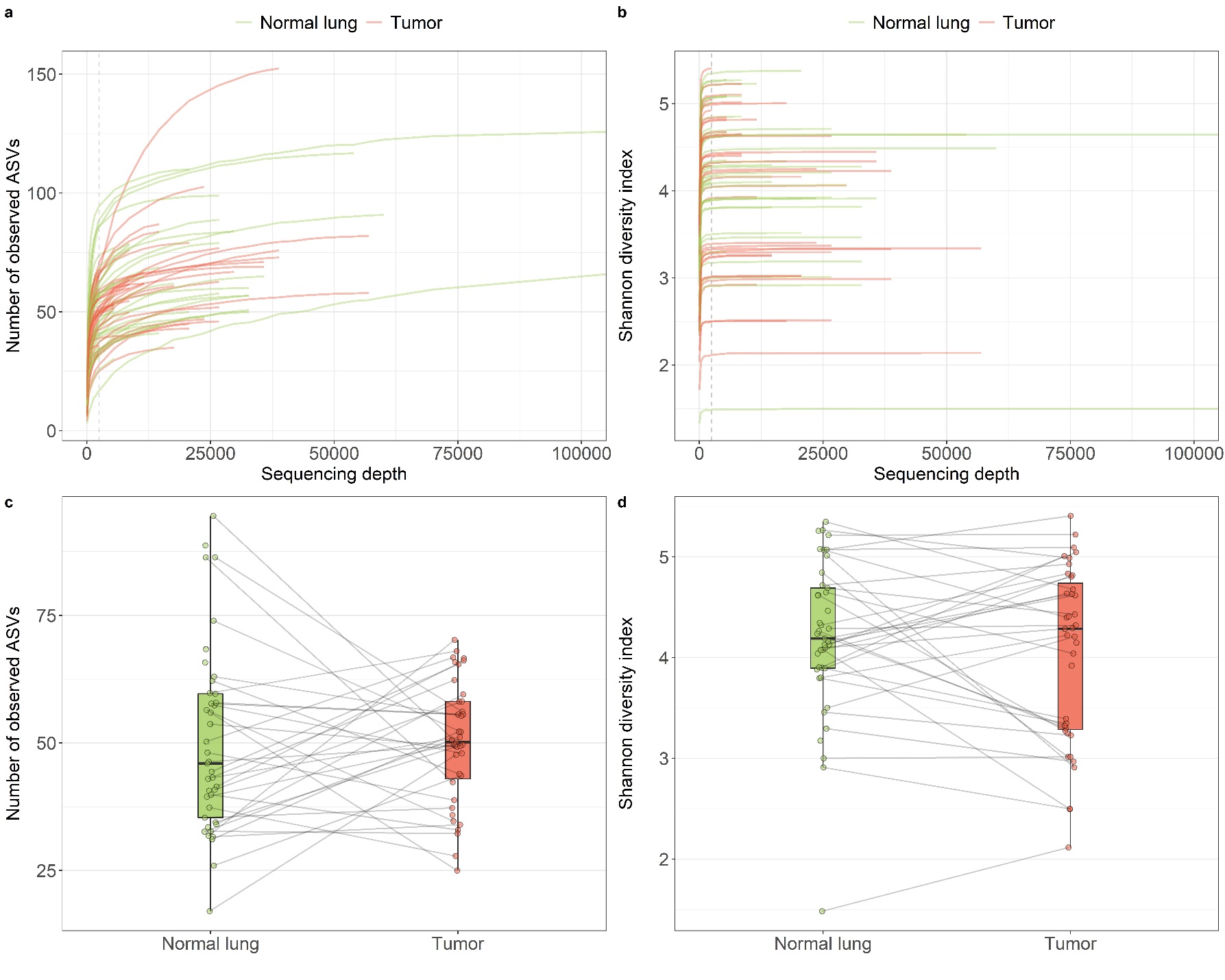


**Supplementary Figure 3.** Rarefaction curves of (a) the number of observed ASVs and (b) the Shannon diversity index. At each depth, the average number of ASVs or Shannon index over 100 iterations was calculated. Vertical dotted line at 2,468 sequence reads represents the lowest depth among the samples after the Deblur workflow, removal of contaminants, and exclusion of samples with low read counts.


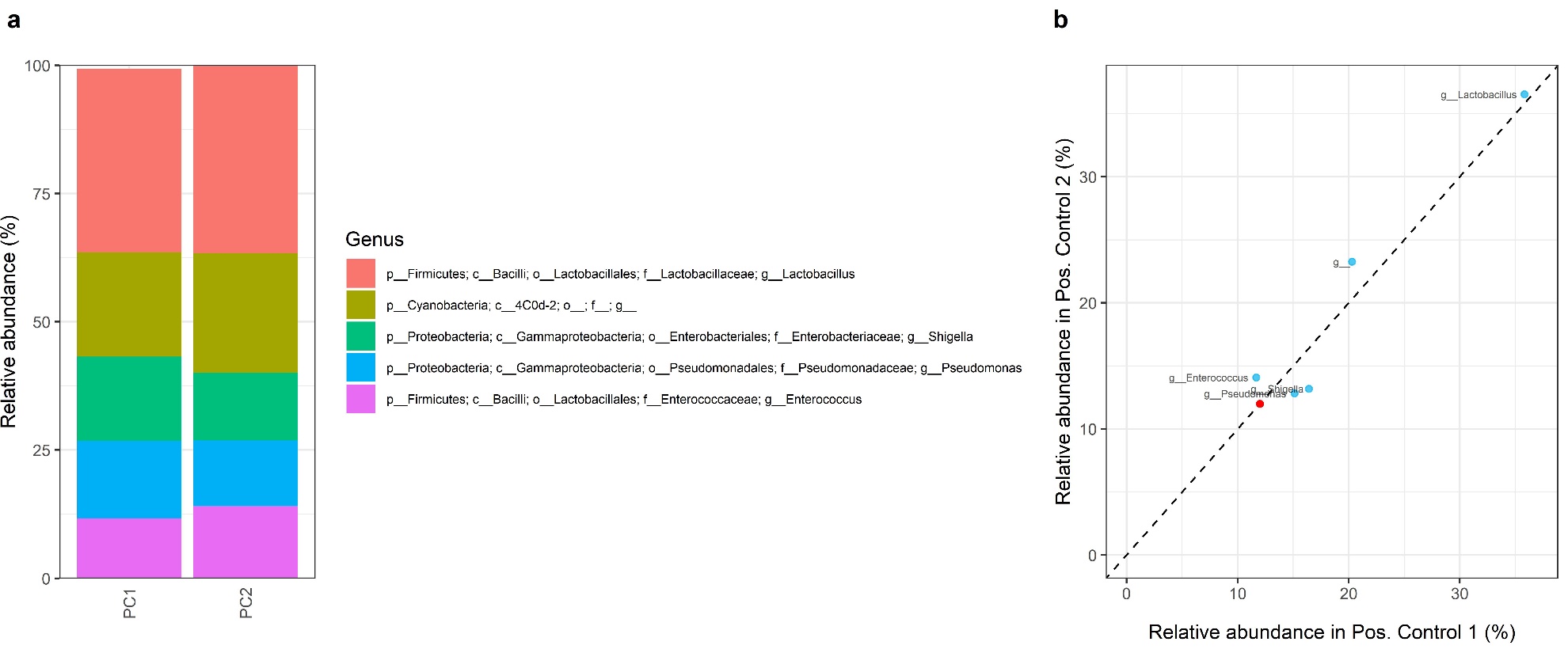


**Supplementary Figure 4.** (a) Relative abundance of genera in the two positive controls (ZymoBIOMICS Microbial Community Standard, Zymo Research, Irvine, CA). Only genera with relative abundance >0.1% are shown. (b) Scatter plot of the relative abundance of these genera in the first and second positive control. The red dot signifies the expected abundance (12%) of the following species: *Listeria monocytogenes, Pseudomonas aeruginosa, Bacillus subtilis, Escherichia coli, Salmonella enterica, Lactobacillus fermentum, Enterococcus faecalis, Staphylococcus aureus*.

**
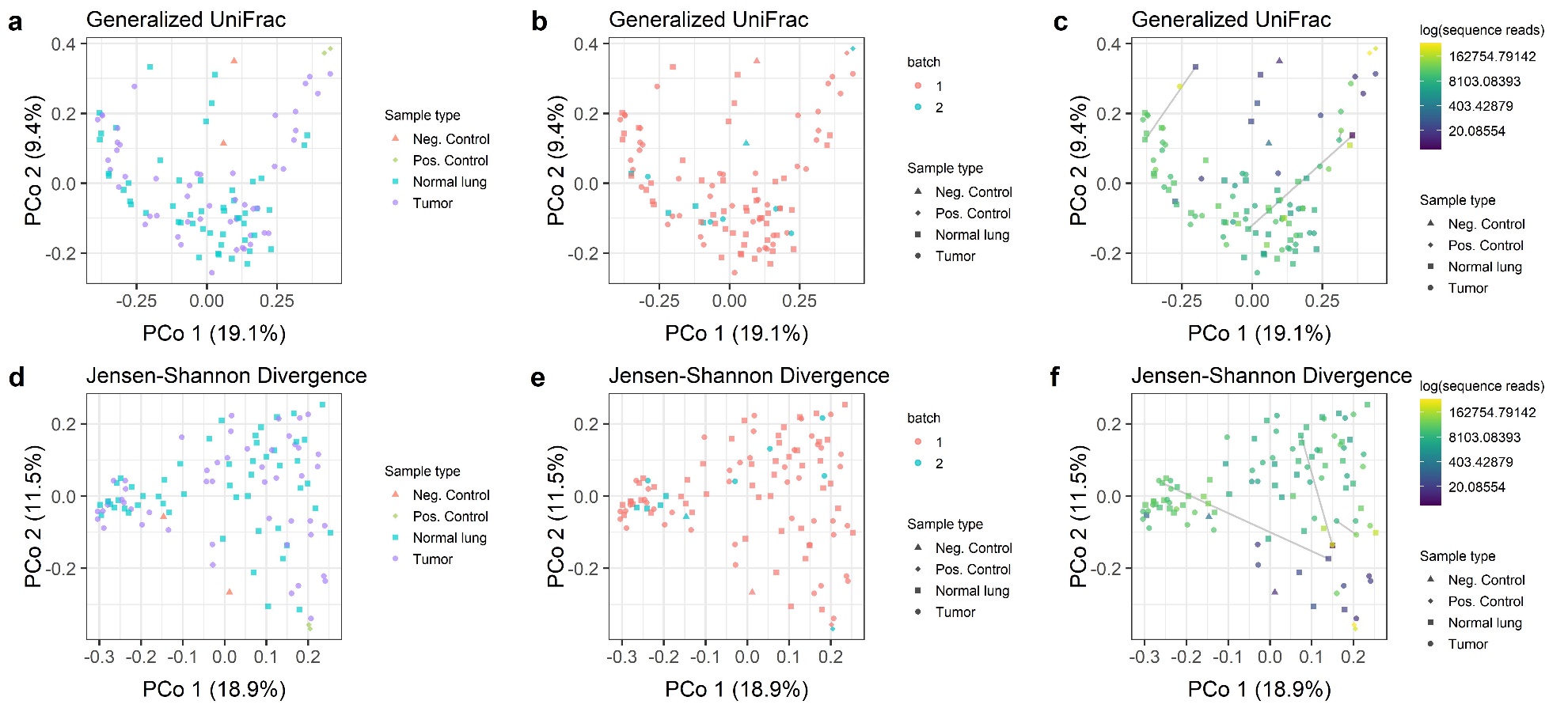
**

**Supplementary Figure 5.** Principal coordinate analysis of (a-c) the generalized UniFrac distance and (d-f) the Jensen-Shannon Divergence. From left to right, plots are colored by sample type, batch, and the number of sequence reads after Deblur and contaminant removal. In the right-most plots, lines connect duplicate samples.

**
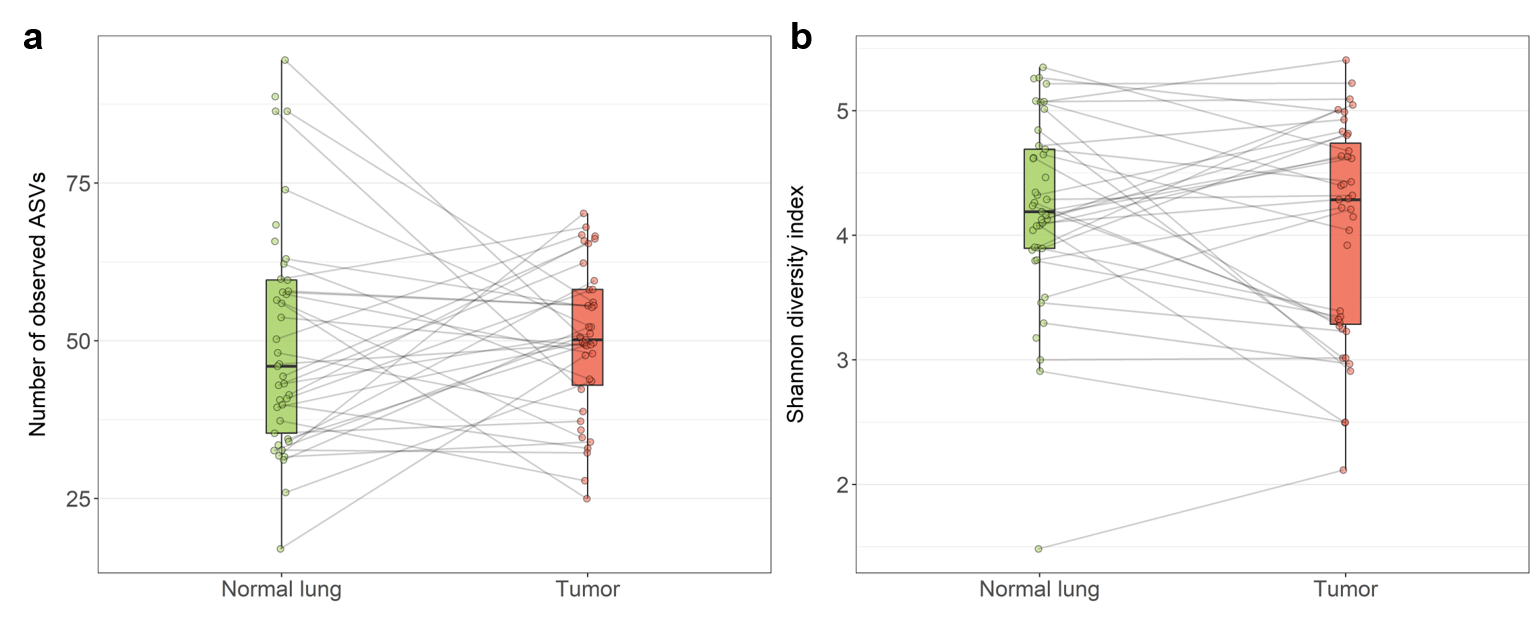
Supplementary Figure 6.** Boxplots of (a) the number of observed ASVs and (b) the Shannon diversity index in tumor and normal lung samples at the lowest sequencing depth (2,468 sequence reads) after the Deblur workflow, contaminant removal, and exclusion of samples with low read counts. Lines connect samples from the same patient.

**
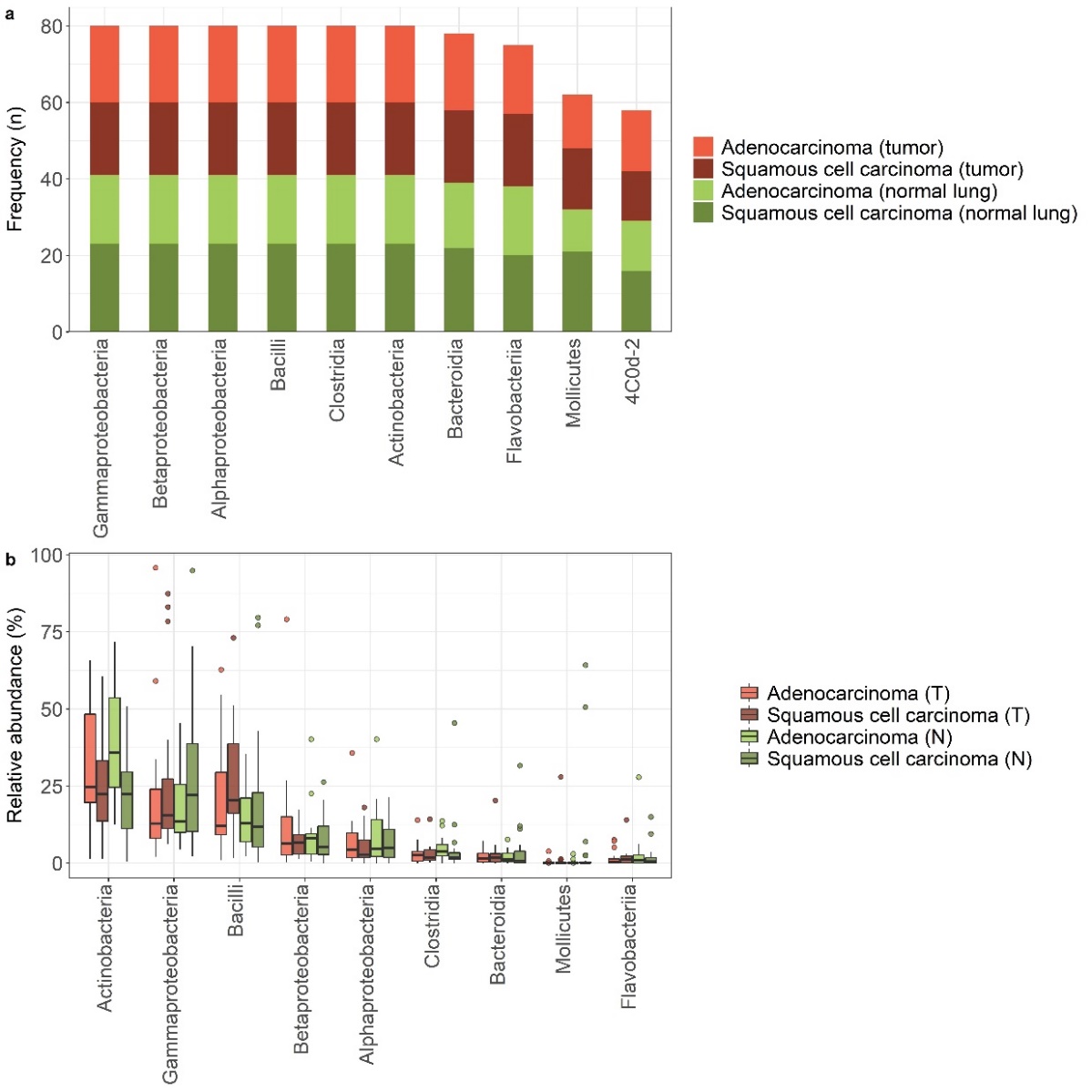
**

**Supplementary Figure 7.** (a) Frequency of common classes in lung samples. Y-axis represents the number of samples (out of 80 samples total). Plot shows all classes present in >50% of the lung samples. (b) Relative abundance of classes in lung samples. Plot shows all classes with mean relative abundance >1%.


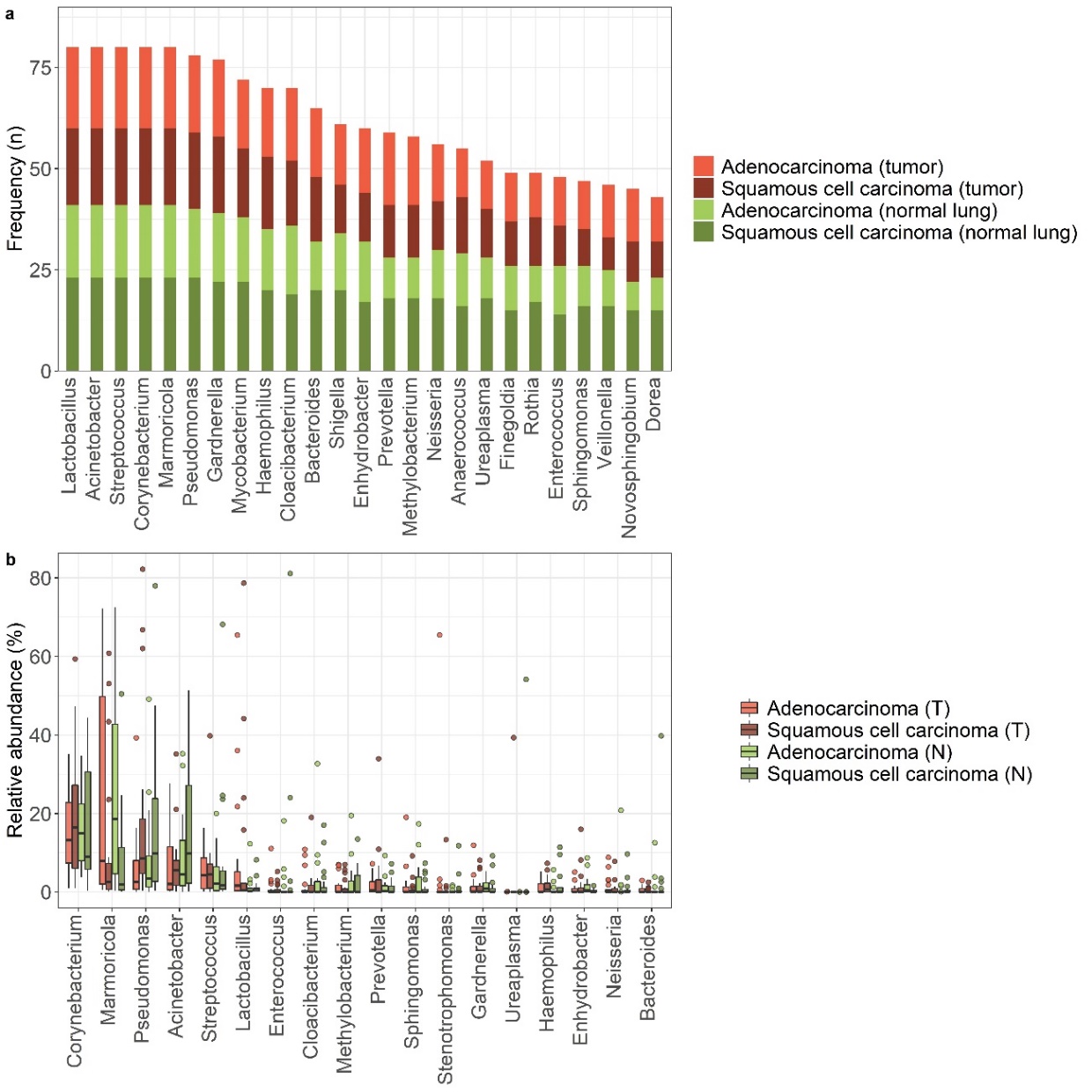


**Supplementary Figure 8.** (a) Frequency of common genera in lung samples. Y-axis represents the number of samples (out of 80 samples total). Plot shows all genera present in >50% of the lung samples. (b) Relative abundance of genera in lung samples. Plot shows all genera with mean relative abundance >1%.


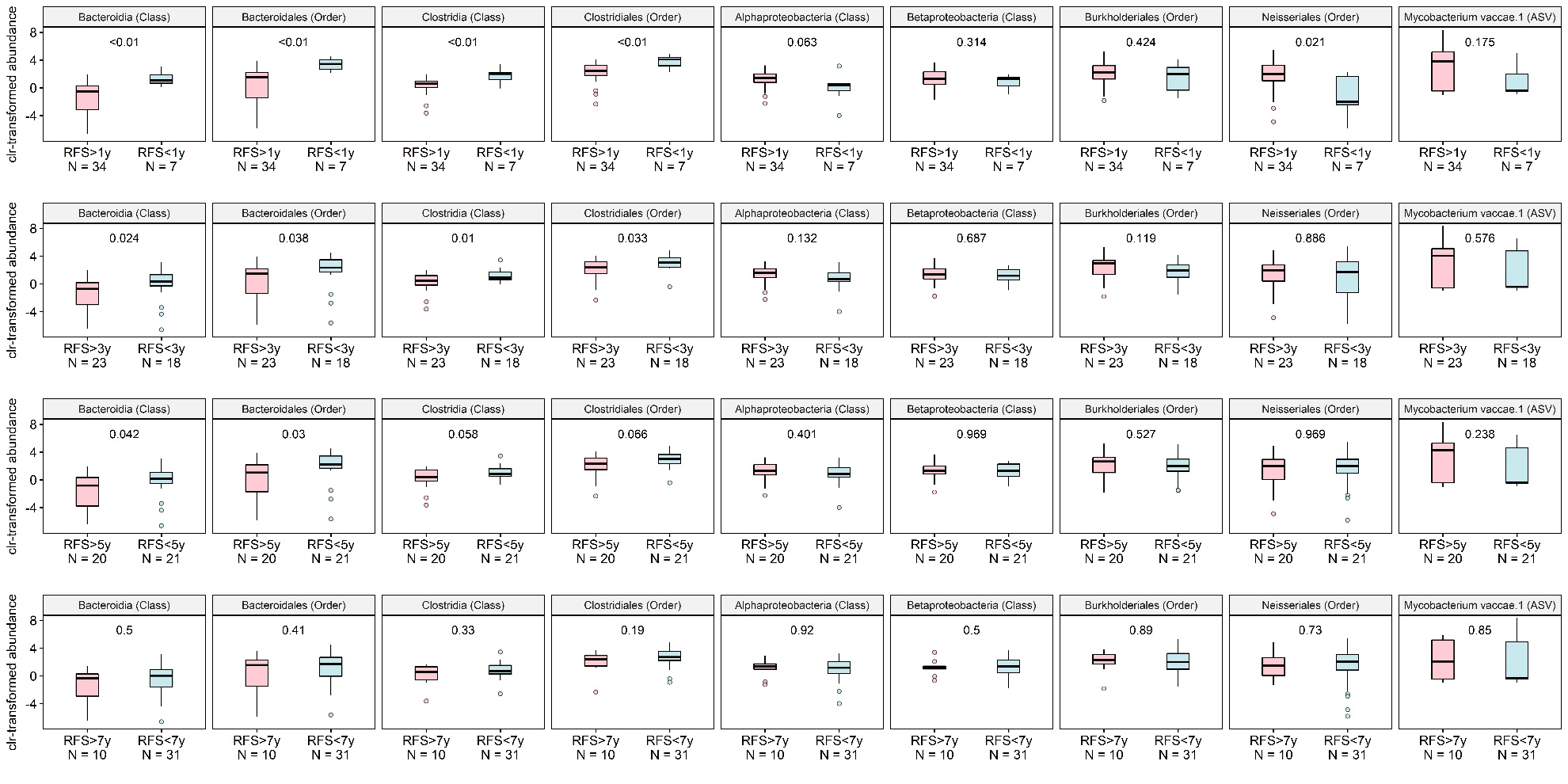


**Supplementary Figure 9.** Boxplots of clr-transformed taxon abundance in normal lung tissue according to 1-year, 3-year, 5-year, and 7-year RFS status. P-values from Wilcoxon rank-sum test.


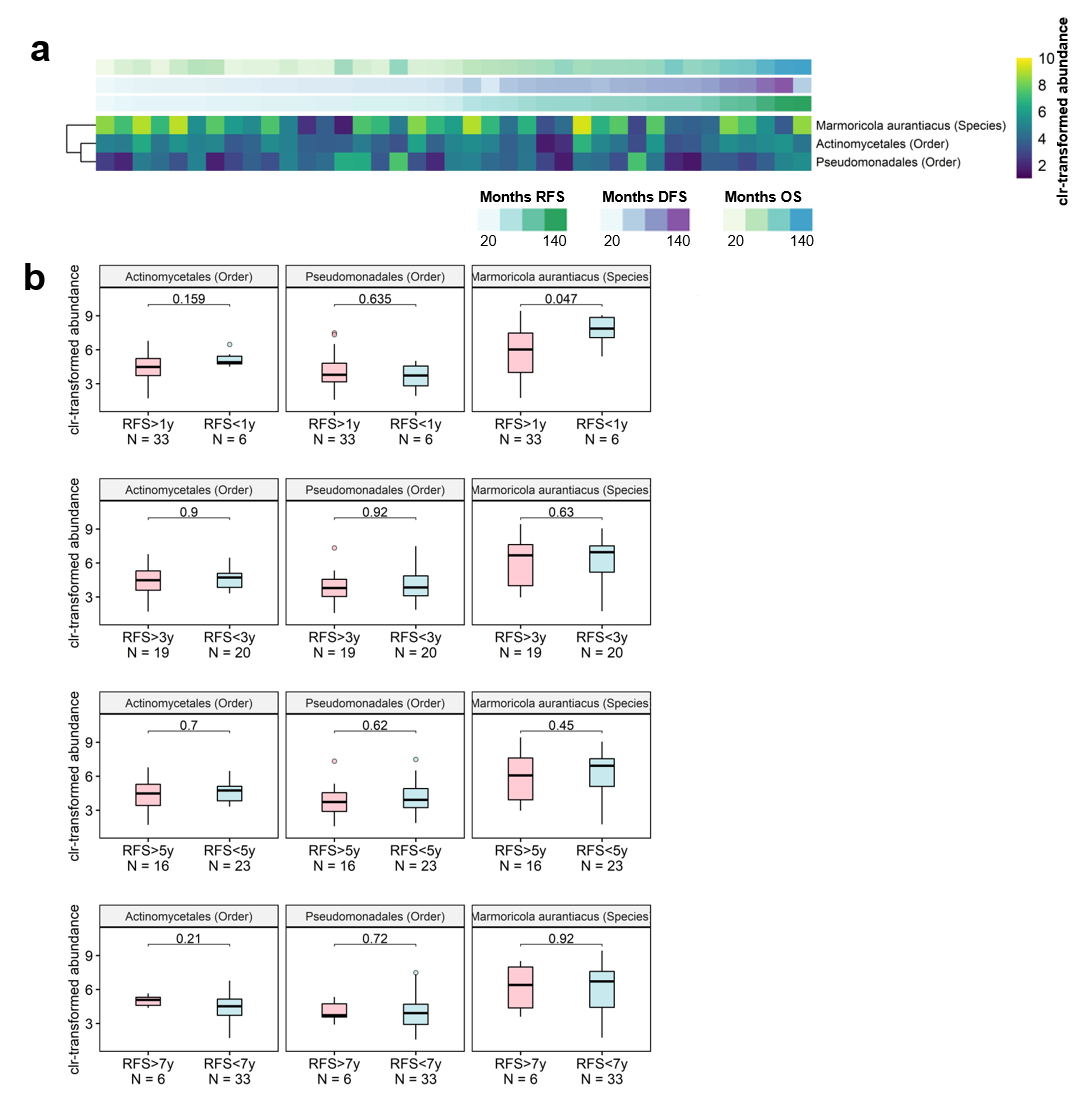


**Supplementary Figure 10.** (a) Heatmap of clr-transformed taxon abundance in normal lung tissue, sorted by months of RFS. (b) Boxplots of clr-transformed taxon abundance in tumor tissue according to 1-year, 3-year, 5-year, and 7-year RFS status. P-values from Wilcoxon rank-sum test.


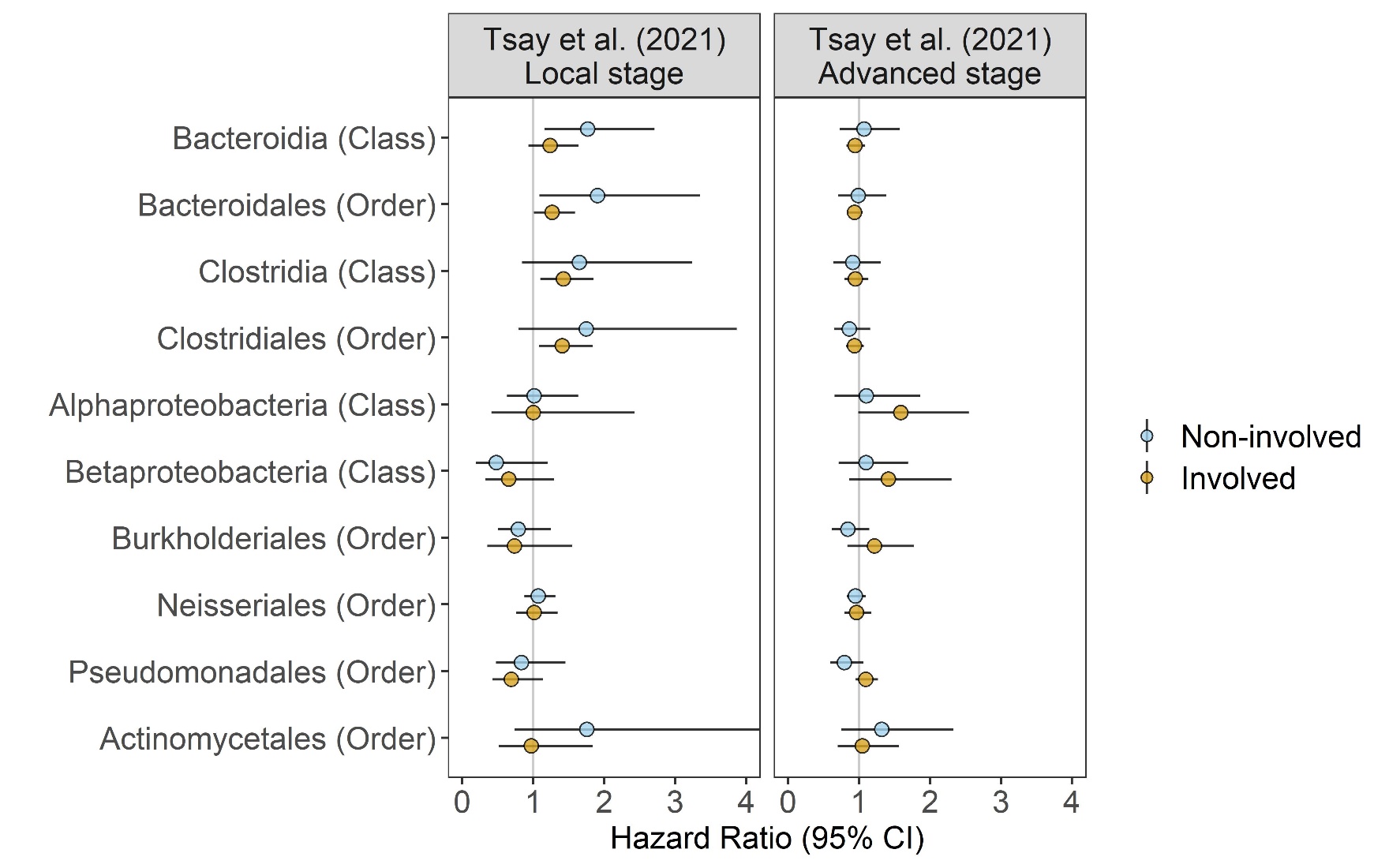
**Supplementary Figure 11.** Plots show the hazard ratio (95% CI) from Cox proportional hazards regression of OS on clr-transformed taxon abundance. Tsay et al. (2021) included lower airway brushings from local stage (I-IIIA) (n=34 patients, n=93 samples [36 involved, 57 non-involved]), and advanced stage (IIIB-IV) (n=39 patients, n=90 samples [40 involved, 50 non-involved]) NSCLC patients. Models were adjusted for age, sex, race, histology, smoking status, and chemotherapy (and surgery in local stage); models also accounted for clustering of samples within patients.

**
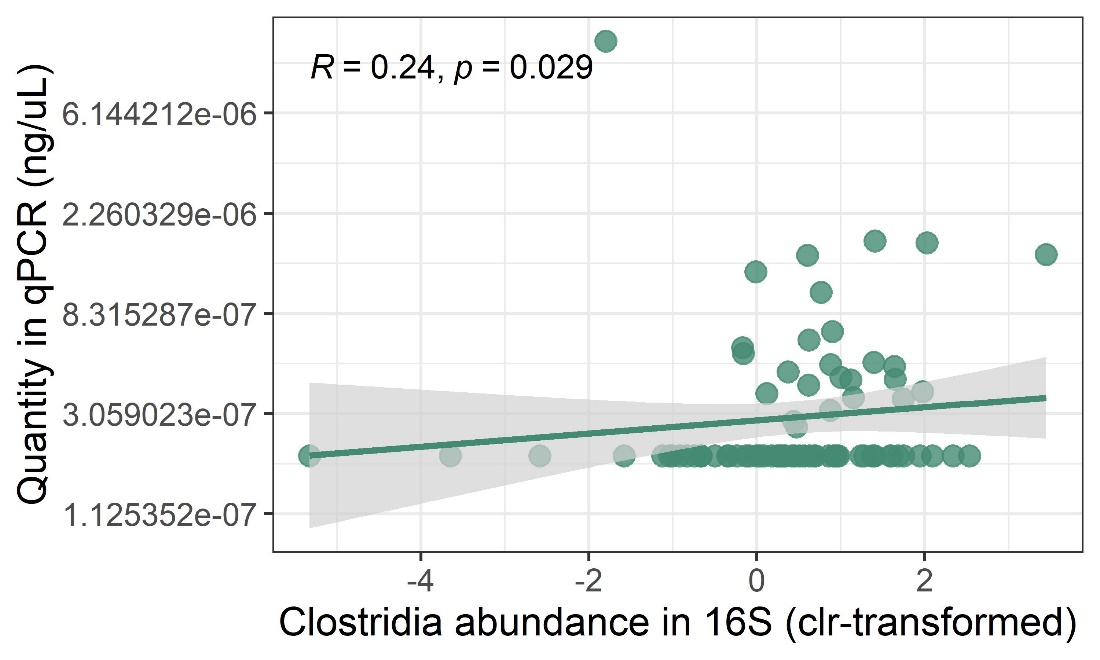
**

**Supplementary Figure 12.** Scatter plot of clr-transformed Clostridia abundance (from 16S data) with qPCR quantity of Clostridia (log-transformed axis). A pseudocount (half the minimum non-zero quantity) was added to the qPCR quantity to allow log transformation. Spearman correlation and p-value are shown on the plot, along with the linear regression line.


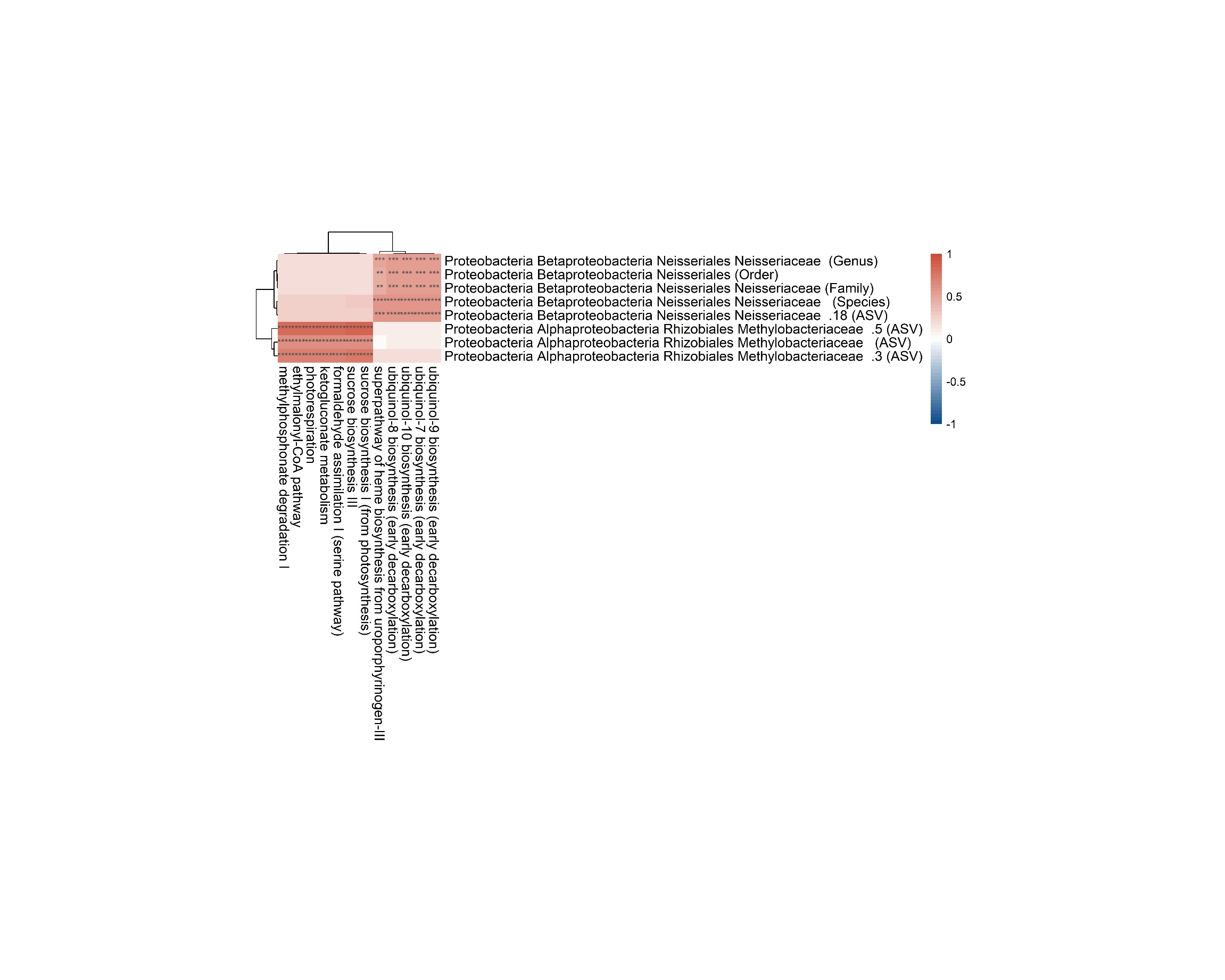


**Supplementary Figure 13.** Spearman’s correlations of taxa associated with survival-related functional pathways in normal lung. Relative abundance of taxa and pathways was used in analysis. Only taxa with correlations ≥0.5 are included in the heatmap. *p<0.05, **p<0.01, ***p<0.001, ****p<0.0001.

**
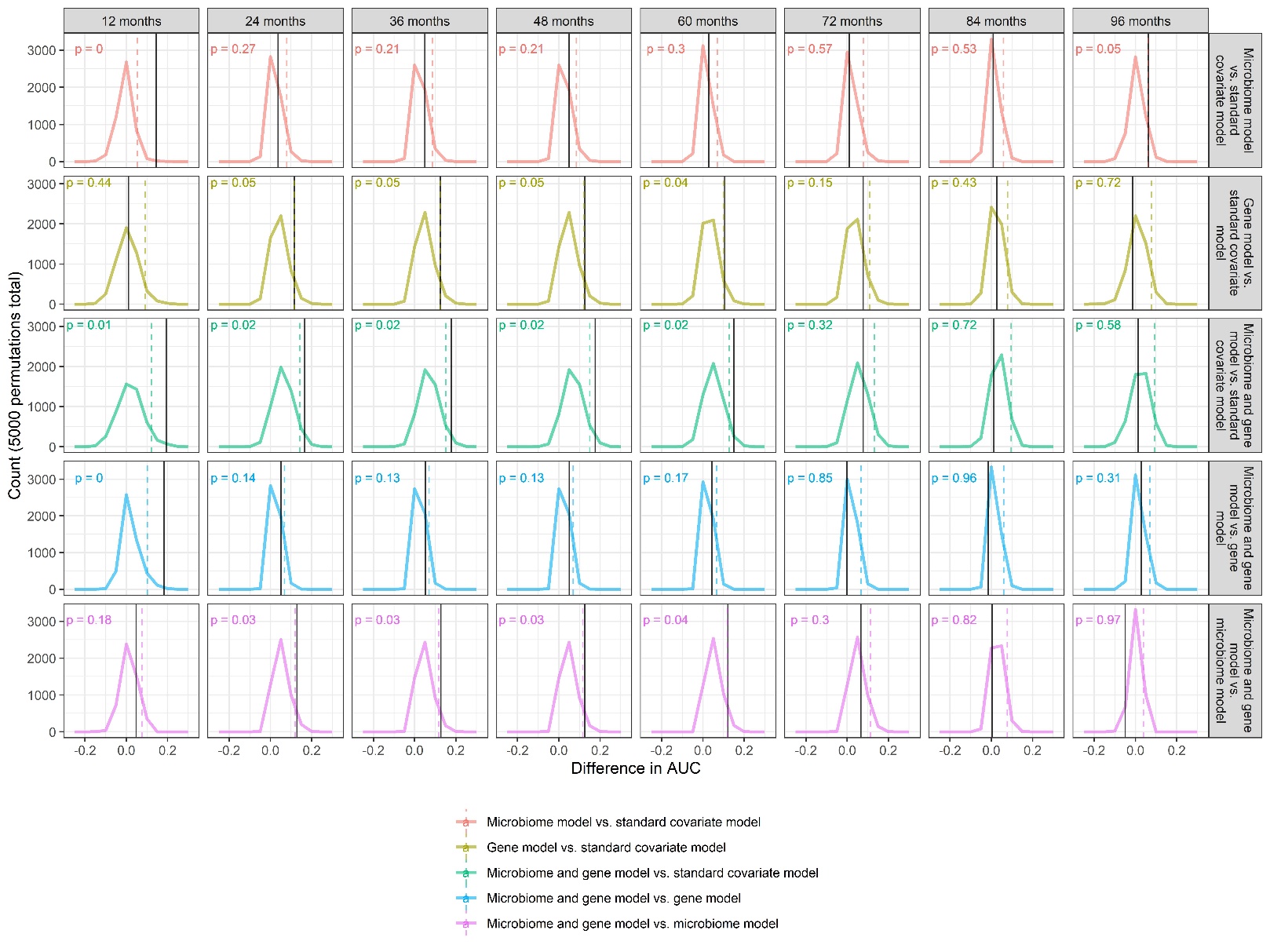
**

**Supplementary Figure 14.** Null distributions of difference in time-dependent AUC for RFS, from 5000 permutations. Solid black line represents the observed (non-permuted) difference in AUC; dotted line represents the 95th percentile of the permuted distribution.

**
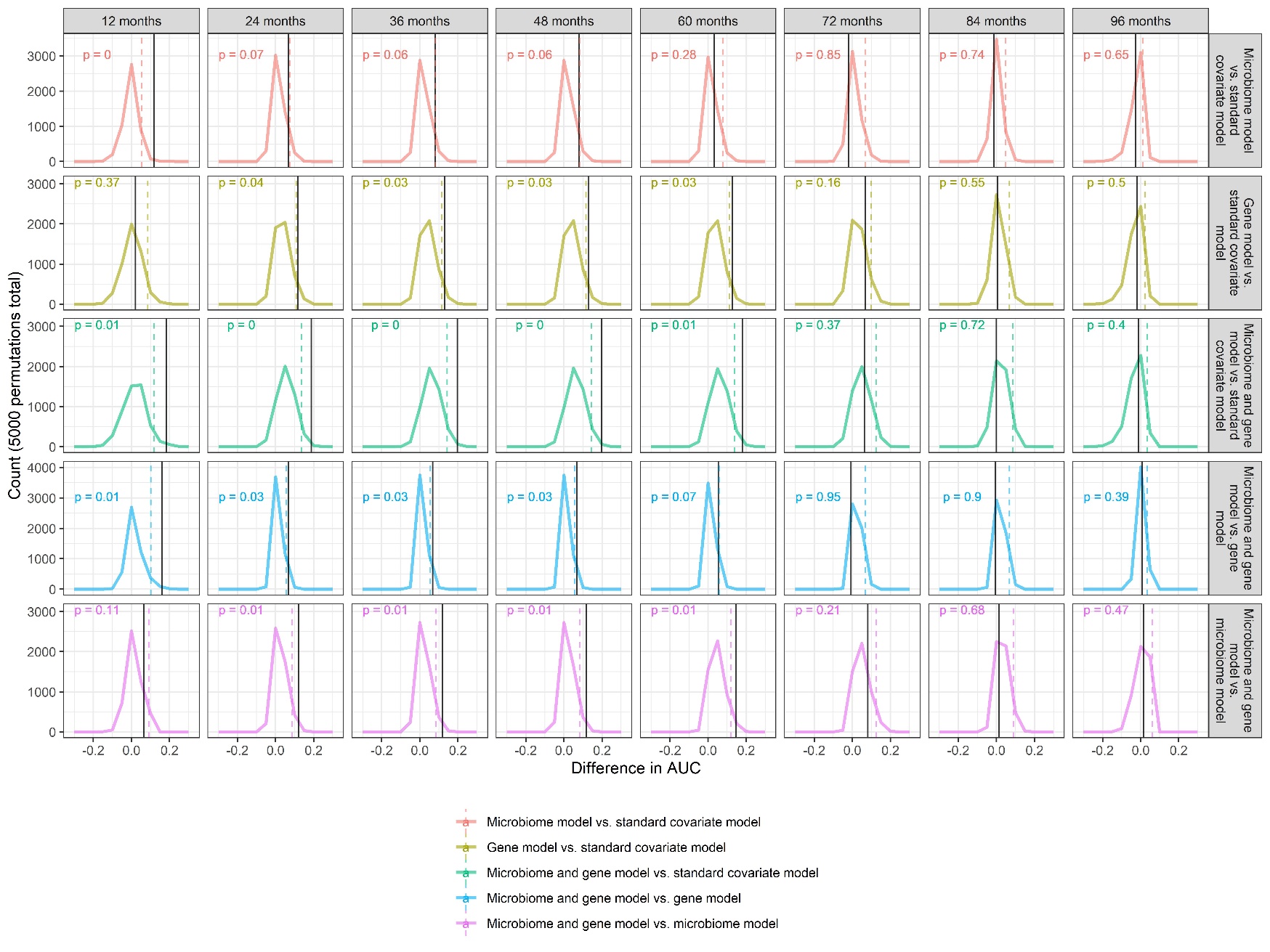
Supplementary Figure 15.** Null distributions of difference in time-dependent AUC for DFS, from 5000 permutations. Solid black line represents the observed (non-permuted) difference in AUC; dotted line represents the 95th percentile of the permuted distribution.
